# Novel BODIPY Dyes with a *Meso*-Benzoxadiazole Substituent: Synthesis, Photophysical Studies, and Cytotoxic Activity Under Normoxic and Hypoxic Conditions

**DOI:** 10.3390/biomedicines13020297

**Published:** 2025-01-25

**Authors:** Weronika Porolnik, Natalia Karpinska, Marek Murias, Jaroslaw Piskorz, Malgorzata Kucinska

**Affiliations:** 1 Chair and Department of Toxicology, Poznan University of Medical Sciences, 3 Rokietnicka Street, 60-806 Poznan, Poland; w.porolnik@op.pl (W.P.); marek.murias@ump.edu.pl (M.M.); 2 Chair and Department of Inorganic and Analytical Chemistry, Poznan University of Medical Sciences, 3 Rokietnicka Street, 60-806 Poznan, Poland; nataliakarpinska20@gmail.com; 3 Doctoral School, Poznan University of Medical Sciences, 70 Bukowska Street, 60-812 Poznan, Poland

**Keywords:** anticancer research, benzofurazan, BODIPY, halogenation, heavy atom effect, hypoxia, oxadiazole, photochemistry, photodynamic therapy, singlet oxygen

## Abstract

**Background/Objectives:** Novel boron dipyrromethene derivatives with a heterocyclic, benzoxadiazole substituent were obtained as potential candidates for the photodynamic therapy (PDT) of cancers. Photochemical properties (e.g., singlet oxygen generation quantum yields (Φ_Δ_), absorption, and emission spectra) and cytotoxic activity studies in normoxic and hypoxic conditions were performed to verify the potential of novel BODIPYs as photosensitizers for PDT. **Methods:** Obtained dyes were characterized using mass spectrometry and various NMR techniques. The relative method with Rose Bengal as a reference and 1,3-diphenylisobenzofuran as a singlet oxygen quencher was used to determine Φ_Δ_ values. The in vitro studies were conducted on human ovarian carcinoma (A2780) and human breast adenocarcinoma (MDA-MB-231) cells. **Results:** Photochemical studies showed that the presence of benzoxadiazole moiety only slightly affected the localization of the absorption maxima but resulted in fluorescence quenching compared with *meso*-phenyl-substituted analogs. In addition, brominated and iodinated analogs revealed a high ability to generate singlet oxygen. Anticancer studies showed high light-induced cytotoxicity of BODIPYs containing heavy atoms with very low IC_50_ values in the 3.5–10.3 nM range. Further experiments revealed that both compounds also demonstrated phototoxic activity under hypoxic conditions. The most potent cytotoxic effect in these conditions was observed in the iodinated BODIPY analog with IC_50_ values of about 0.3 and 0.4 μM for A2780 and MDA-MB-231 cells, respectively. **Conclusions:** The results of this study highlighted the advantages and some potential drawbacks of BODIPY compounds with heavy atoms and benzoxadiazole moiety as a useful scaffold in medicinal chemistry for designing new photosensitizers.

## 1. Introduction

Boron dipyrromethene derivatives (BODIPYs) and their analogs with the carbon atom at 8 position (*meso*) replaced by nitrogen (aza-BODIPY) are important dyes with numerous applications, including photovoltaics, catalysis, fluorescence imaging, and the preparation of various sensors and optical devices [1,2]. BODIPY dyes have been commonly used as fluorescent markers for various biomedical applications, including imaging, disease diagnosis, and biological process monitoring. They have also been utilized for anticancer treatment as sensitizers in photodynamic therapy (PDT) and photothermal therapy (PTT) [3]. BODIPY dyes are also connected with other fluorophores, such as xanthenes, cyanines, and coumarins, which are used as fluorophores for various biological applications [4]. In addition, BODIPYs are promising building blocks for various materials for fluorescence labeling and detection, photoacoustic imaging, photodynamic and photothermal therapy, and theranostic agents for integrated diagnosis and treatment [5]. Special attention is paid to using BODIPY dyes in PDT [6,7]. This light-activated therapy proved highly efficient against various cancers, microbial infections, and some non-cancerous diseases [8,9]. The mechanism of photodynamic action usually involves the presence of molecular oxygen in the treated environment to produce reactive oxygen species. For this reason, commonly occurring tumors in oxygen deficient, hypoxic conditions are one of the most critical challenges of PDT [10,11].

BODIPY photosensitizers have been extensively studied for many years, and various modifications have been tested to improve their physicochemical and photochemical properties [12]. Heavy atom substitution is one of the most effective modifications for enhancing singlet oxygen quantum yield. The presence of heavy atoms increases the probability of singlet to triplet intersystem crossing by quenching fluorescence and enhancing spin–orbit coupling [6,13]. In our previous work, we demonstrated the activity of BODIPYs substituted at the *meso* position, with N, N-dimethylaminopropoxyphenyl moiety and with the iodine atoms in positions 2 and 6 of the BODIPY’s core. We found that the presence of iodine can indeed increase singlet oxygen generation, from 0.02 for a heavy metal-free BODIPY to 0.97 for iodinated derivatives. Furthermore, inserting two iodine atoms can decrease IC_50_ values from ~3 µM to 48 nM [14].

Boron dipyrromethene dyes are commonly substituted at the *meso* position with phenyl or alkyl groups [3,7,15]. It is known that the substitution at the *meso* position of the BODIPY scaffold can change the electronic density upon excitation and modify the compound’s properties [16]. The *meso*-substituted phenyl with an electron donor (e.g., amino-phenyl) and electron-withdrawing substituents (e.g., nitro or carboxyl group) can change the physicochemical and photochemical properties, including fluorescence emission, singlet oxygen generation, lipophilicity, and solubility [16,17,18,19,20]. Heterocyclic substituents at *meso* positions are less common, but recently reported examples have shown interesting properties and possible biomedical applications. Shi et al. synthesized *meso*-benzothiazole BODIPYs as fluorescent rotors for sensing lysosomal viscosity changes, which can be used to monitor the autophagy process and related diseases [21]. A *meso*-indol-substituted BODIPY showed high photocytotoxicity against human lung adenocarcinoma A549 cells and was also used for two-photon fluorescence imaging performed in zebrafish [22]. Several BODIPY dyes with heterocyclic substituents such as thiophene, thiazole, and thienothiophene at the *meso* position have been prepared and subjected to antiparasitic studies in *Trypanosoma brucei* and *Leishmania major* [23]. In addition, BODIPYs with *meso*-benzodioxole substituent revealed promising photodynamic inactivation of the *Staphylococcus aureus* bacteria [24].

Oxadiazoles are five-membered heterocycles containing one oxygen and two nitrogen atoms existing in different isomeric forms. There are many examples of 1,2,4- and 1,3,4-oxadiazoles, while 1,2,5-oxadiazoles, also known as furazans, are less common but have recently gained more attention. In addition, furazans can form fused, bicyclic rings such as benzofurazan. Benzofurazan is a chemical scaffold that can balance the lipophilic character with a high degree of polarization and inductive effect, ensured by the oxadiazole moiety. Additionally, furazan itself exhibits a significant electron-withdrawing characteristic [25]. Various chemical and biological applications of furazan-based compounds, including fluorescent probes for biomolecules, as well as antimicrobial and anticancer agents, have been researched. In addition, benzofurazan moiety is found in the structure of isradipine, a dihydropyridine calcium channel antagonist utilized to treat hypertension [25,26]. The derivatives of nitrobenzoxadiazole (nitrobenzofurazan, NBD) have attracted particular attention due to their fluorescent properties, which can be used to label various important molecules in medicinal chemistry and biology [27].

A few examples of dyes from the boron dipyrromethene class possess oxadiazole moiety. Two BODIPYs with one or two NBD substituents linked via styryl group were synthesized as fluorescence probes for hydrogen sulfide (H_2_S) detection. These probes were successfully used for hydrogen sulfide imaging in cultured cells and zebrafish [28]. Another fluorescent probe for H_2_S, containing an NBD substituent, was synthesized by Ding et al. The broad studies using aqueous solutions, human cultured cells, and zebrafish showed good characteristics for detecting hydrogen sulfide, including a fast response with intensive emission at 580 nm, high selectivity, low detection limit, and low cytotoxicity [29]. NBD moiety was also introduced to the aza-BODIPY dye to obtain the near-infrared fluorescent probe for discriminative detection of cellular thiols, such as cysteine, homocysteine, and glutathione in living cells [30]. A BODIPY dye containing amino-nitro-benzofurazan moiety was used to simultaneously generate singlet oxygen and nitric oxide. The efficient light-induced production of two independent cytotoxic agents and efficient encapsulation in biodegradable nanoparticles make this molecule a promising candidate for the photodynamic treatment of cancers [31]. It is worth mentioning that to the best of our knowledge, there are no BODIPYs with oxadiazole or benzoxadiazole moieties attached directly at the *meso* position.

**Figure 1:**
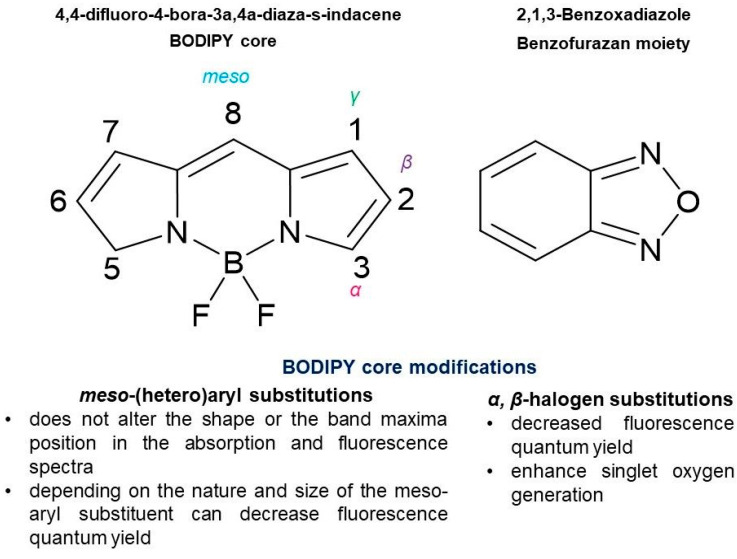
The chemical scaffolds used in this study. The figure also illustrates the possibilities of chemical modification involving the incorporation of aryl-substituent at the *meso* position and halogenation in the BODIPY core.

The presented studies describe novel BODIPYs with heterocyclic, benzoxadiazole substituents at the *meso* position. Photochemical studies and in vitro cytotoxic activity assays towards two human cancer cell lines under normoxic and hypoxic conditions were performed to verify the potential of novel BODIPYs as photosensitizers for anticancer PDT. The chemical scaffolds used in this study and the effect of modifications involving substitution at the *meso* position and halogenation in the BODIPY structure [32] are presented in Figure 1.

## 2. Materials and Methods

### 2.1. General Procedures

Reactions were conducted under an argon atmosphere on a heat gun-dried glass with Heat-On heating blocks (Radleys, Essex, UK). Aluminum-backed silica gel plates with an F-254 indicator (SiliCycle, Quebec, QC, Canada) were used for thin-layer chromatography (TLC). Solvents were removed at reduced pressure using the R-100 (Buchi Labortechnik, Flawil, Switzerland) and Hei-VAP (Heidolph Instruments, Schwabach, Germany) rotary evaporators. Absorption spectra were acquired with a Shimadzu UV-1900i spectrophotometer (Shimadzu Deutschland, Duisburg, Germany). The AvanceCore 400 spectrometer (Bruker, Billerica, MA, USA) at the Center of Innovative Pharmaceutical Technology at Poznan University of Medical Sciences was utilized for nuclear magnetic resonance spectroscopy (NMR). The ^1^H spectra were recorded at 400.13 MHz, while ^13^C at 100.61 MHz. The chemical shift values (*δ*) were referred to the solvent residual peak (CDCl_3_) and given as parts per million (ppm). The singlet and doublet of doublet signals were observed and abbreviated as s and dd, respectively. The QTOF Impact HD spectrometer (Bruker, Billerica, MA, USA) at the Center for Advanced Technologies (Adam Mickiewicz University in Poznan, Poland) was used to acquire high-resolution mass spectra (HRMS). The *m*/*z* values were presented with the intensity as a percentage of the maximal signal and the difference (Δ ppm) between the identified and calculated mass.

### 2.2. Synthetic Procedures

#### 2.2.1. Compound **1**, 8-(2,1,3-Benzoxadiazol-4-yl)-1,3,5,7-tetramethyl-4,4’-difluoro-4-bora-3a,4a-diaza-s-indacene

Two drops of trifluoroacetic acid were added to the mixture of 4-formyl-2,1,3-benzoxadiazole (1 mmol, 150 mg) and 2,4-dimethylpyrrole (2 mmol, 0.21 mL) in 50 mL of anhydrous dichloromethane. After stirring at room temperature for 1 h, DDQ (2 mmol, 450 mg) in dichloromethane (25 mL) was added, and the reaction was carried out at room temperature for another hour. Next, N,N-diisopropylethylamine (15 mmol, 2.61 mL) was added, and then boron trifluoride etherate (19 mmol, 2.47 mL) was instilled, and the reaction mixture was stirred for 72 h. After that, purified water (100 mL) was added, and the obtained mixture was filtrated through Celite and extracted. The organic layer was collected, washed with purified water, and dried with anhydrous sodium sulfate. After the evaporation of the solvent, the solid residue was chromatographed in hexane-dichloromethane 1:2 (*v*/*v*). The obtained product was crystallized from dichloromethane-*n*-pentane to produce a red powder of **1** (560 mg, 31%); R_f_ (dichloromethane: hexane, 1:1 *v*/*v*) = 0.15. HRMS (ESI) *m*/*z*; found [M+H]^+^ 367.1527—5% (4.1 ppm); C_19_H_18_BF_2_N_4_O requires [M+H]^+^ 367.1542; found [M+Na]^+^ 389.1359—100% (0.5 ppm); C_19_H_17_BF_2_N_4_ONa requires [M+Na]^+^ 389.1361; found [M+K]^+^ 405.1090—10% (2.7 ppm); C_19_H_17_BF_2_N_4_OK requires [M+K]^+^ 405.1101. ^1^H NMR (400.1 MHz, CDCl_3_): δ_H_, ppm 8.01 (dd, ^3^J = 9 Hz, 1 Hz, 1H, ArH), 7.56 (dd, ^3^J = 9 Hz, 7 Hz, 1H, ArH), 7.41 (dd, ^3^J = 6.5 Hz, 1 Hz, 1H, ArH), 6.00 (s, 2H, H-pyrrole), 2.57 (s, 6H, -CH_3_), 1.30 (s, 6H, -CH_3_). ^13^C NMR (100.6 MHz, CDCl_3_): δ_C_, ppm 14.5; 14.7; 117.6; 121.9; 124.7; 131.2; 131.3; 131.3; 131.6; 133.1; 141.9; 148.8; 149.0; 157.0.

#### 2.2.2. Compound **2a**, 8-(2,1,3-Benzoxadiazol-4-yl)- 2,6-dibromo-1,3,5,7-tetramethyl-4,4’-difluoro-4-bora-3a,4a-diaza-s-indacene

BODIPY **1** (146.5 mg, 0.4 mmol) was added to 30 mL of dichloromethane. Next, N-bromosuccinimide (213.6 mg, 1.2 mmol) dissolved in dichloromethane (10 mL) was instilled into the reaction mixture. After 4 h, the solvent was evaporated, and the solid residue was chromatographed, with dichloromethane: hexane 1:5 (*v*/*v*) as the eluent. Precipitation from a dichloromethane solution with *n*-pentane gave a dark powder of **2a** (145 mg, 69%). R_f_ (dichloromethane: hexane, 1:1 *v*/*v*) = 0.53. HRMS (ESI) *m*/*z*; found [M-F]^+^ 502.9674—0.5% (3.2 ppm); C_19_H_15_BBr_2_FN_4_O requires [M-F]^+^ 502.9690; found [M+H]^+^ 522.9729—0.3% (4.4 ppm); C_19_H_16_BBr_2_F_2_N_4_O requires [M+H]^+^ 522.9752; found [M+Na]^+^ 544.9562—50% (1.7 ppm); C_19_H_15_BBr_2_ F_2_N_4_ONa requires [M+Na]^+^ 544.9571; found [M+K]^+^ 560.9295—4.3% (2.9 ppm); C_19_H_15_ Br_2_BF_2_N_4_OK requires [M+K]^+^ 560.9311. ^1^H NMR (400.1 MHz, CDCl_3_): δ_H_, ppm 8.07 (dd, ^3^J = 9 Hz, 1 Hz, 1H, ArH), 7.59 (dd, ^3^J = 9 Hz, 7 Hz, 1H, ArH), 7.41 (dd, ^3^J = 7 Hz, 1 Hz, 1H, ArH), 2.63 (s, 6H, -CH_3_), 1.30 (s, 6H, -CH_3_). ^13^C NMR (100.6 MHz, CDCl_3_): δ_C_, ppm 13.9; 14.0; 112.7; 118.4; 124.2; 130.3; 131.4; 131.9; 133.6; 139.5; 148.7; 149.1; 155.7.

#### 2.2.3. Compound **2b**, 8-(2,1,3-Benzoxadiazol-4-yl)- 2,6-diiodo-1,3,5,7-tetramethyl-4,4’-difluoro-4-bora-3a,4a-diaza-s-indacene

BODIPY **1** (146 mg; 0.4 mmol) was dissolved in anhydrous ethanol (10 mL), and iodine (152 mg; 0.6 mmol) was added. Next, the iodic acid solution (141 mg; 0.8 mmol) in water (1 mL) was instilled, and the reaction mixture was stirred at 70 °C. The consumption of the substrate was evaluated using TLC, and after 45 min, the solvent was evaporated. The solid residue was chromatographed using silica gel, with dichloromethane: hexane 1:5 to 2:1 *v*/*v* as the eluent. The obtained product was crystallized from dichloromethane-*n*-pentane to give dark crystals of **2b** (196 mg; 79%) R_f_ (dichloromethane: hexane, 1:1 *v*/*v*) = 0.38. HRMS (ESI) *m*/*z*; found [M−F]^+^ 598.9404—0.8% (1.3 ppm); C_19_H_15_BFBr_2_N_4_O requires [M−F]^+^ 598.9412; found [M+H]^+^ 618.9469—0.7% (0.8 ppm); C_19_H_16_BBr_2_F_2_N_4_O requires [M+H]^+^ 618.9474; found [M+Na]^+^ 640.9296—62% (0.3 ppm); C_19_H_15_BF_2_I_2_N_4_ONa requires [M+Na]^+^ 640.9294; found [M+K]^+^ 656.9039—100% (0.8 ppm); C_19_H_15_BF_2_I_2_N_4_OK requires [M+K]^+^ 656.9034. ^1^H NMR (400.1 MHz, CDCl_3_): δ_H_, ppm 8.07 (dd, ^3^J = 9 Hz, 1 Hz, 1H, ArH), 7.59 (dd, ^3^J = 9 Hz, 7 Hz, 1H, ArH), 7.40 (dd, ^3^J = 7 Hz, 1 Hz, 1H, ArH), 2.67 (s, 6H, -CH_3_), 1.32 (s, 6H, -CH_3_). ^13^C NMR (100.6 MHz, CDCl_3_): δ_C_, ppm 16.4; 17.2; 86.7; 118.3; 124.6; 131.2; 131.4; 131.8; 132.8; 144.2; 148.7; 149.1; 158.4.

### 2.3. Absorption and Emission Properties

The UV-Vis spectra of BODIPYs **1**, **2a**, and **2b** were recorded on a Shimadzu UV-1900i spectrophotometer (Shimadzu Deutschland, Duisburg, Germany), while emission spectra were recorded using a Jasco 6200 spectrofluorimeter (Jasco, Tokyo, Japan). The absorption measurements were performed in triplicate, with molar absorption coefficients given as mean values ± SD. The fluorescence quantum yield measurements were conducted in methanol solutions using a fluorescein solution in 0.1 M NaOH (Φ_Fref_ = 0.92 [33]) as a reference for BODIPY **1** and the Rhodamine B solution in ethanol (Φ_Fref_ = 0.97 [34]) in the cases of BODIPYs **2a** and **2b** [35,36,37]. The diluted solutions of BODIPYs and references were excited at 500 nm for BODIPY **1** and 530 nm for **2a** and **2b**. The fluorescence quantum yield (Φ_F_) values were calculated following previously elaborated procedures [17,38] from three experiments and presented as mean ± SD.

### 2.4. Photosensitized Production of Singlet Oxygen

The singlet oxygen generation quantum yields (Φ_Δ_) were established using the relative chemical trapping method [17,35,39], with Rose Bengal as a reference (Φ_ΔMeOH_ = 0.76 [40]) and 1,3-diphenylisobenzofuran (DPBF, Aldrich) as a singlet oxygen quencher. The concentration of DPBF was set to about 3 × 10^−5^ M, and its decomposition was monitored as absorbance decreased at 409 nm. The methanol solutions of BODIPYs **1**, **2a**, and **2b** or Rose Bengal and DPBF in 1 cm quartz cells were exposed to light at 520 nm from the xenon arc lamp and a M250/1200/U monochromator (Optel, Opole, Poland). The light intensity measured using a PM100D meter with an S120VC sensor (Thorlabs GmbH, Bergkirchen, Germany) equaled 0.5 mW/cm^2^. The UV-Vis spectra were recorded at the specific time points of irradiation using a Shimadzu UV-1900i spectrophotometer to determine the rate of DPBF oxidation upon interaction with singlet oxygen generated from tested BODIPYs and Rose Bengal. The Φ_Δ_ values were calculated following previously elaborated procedures [17,38] and presented as mean values from three measurements ± SD.

### 2.5. Cytotoxic Activity

#### 2.5.1. Cell Culture

The reagents used for in vitro studies were obtained from Sigma Aldrich (St. Louis, MO, USA) and included Dulbecco’s modified Eagle’s medium (DMEM), fetal bovine serum (FBS), a penicillin–streptomycin–L-glutamine solution, Dulbecco’s phosphate-buffered saline (DPBS), trypsin–EDTA, molecular-grade dimethyl sulfoxide (DMSO), and 3-(4, 5-dimethylthiazol-2-yl)-2,5-diphenyltetrazolium bromide (MTT).

The DMSO used as a solvent for formazan crystals was purchased from Avantor Performance Materials (Gliwice, Poland).

The human ovarian carcinoma cell line (A2780) and human breast adenocarcinoma (MDA-MB-231) were purchased from the European Collection of Authenticated Cell Cultures (ECACC, Salisbury, UK) and American Type Culture Collection (ATCC, Manassas, VA, USA), respectively. Both cell lines were cultured in DMEM supplemented with 10% (*v*/*v*) FBS, 2 mM L-glutamine, 1% (*v*/*v*) penicillin (10,000 U), and a streptomycin solution (10 mg/mL) in a humidified atmosphere of 5% CO_2_ at 37 °C.

The tested compounds were dissolved in DMSO at a concentration of 10 mM for the preliminary study. In contrast, to determine the IC_50_ values, the compounds were dissolved at a concentration of 1 mM. Stock solutions were stored in the dark at −20 °C. The cytotoxicity of each photosensitizer was evaluated under both dark and light conditions.

Hypoxia experiments were conducted using the hypoxia station (Whitley H35 Hypoxystation; Don Whitley Scientific, Bingley, UK). The hypoxia station ensured stable and monitored experimental conditions by maintaining oxygen at 1%, carbon dioxide at 5%, nitrogen at 94%, a humidified atmosphere, and a temperature of 37 °C. All reagents used for hypoxia experiments were incubated under hypoxic conditions before being added to the cells.

#### 2.5.2. Cell Viability Under Normoxic and Hypoxic Conditions

In the preliminary experiments conducted under normoxic and hypoxic conditions, the cytotoxic potential of the tested compounds was evaluated on MDA-MB-231 cells (seeded at a density of 15 × 10^3^ cells per well)at concentrations of 0.1 µM, 1 µM, and 10 µM. As a negative control, DMSO was used at a concentration of 0.1% (*v*/*v*) in a cell culture medium. Cells were treated with compounds for 24 h. Following irradiation, the cells were washed twice with DPBS and irradiated at a wavelength of 525 nm with InGaN-based High Power LED Multi-Chip Emitters (RoithnerLaserTechnik, Vienna, Austria). The PM16-130 power meter with a slim photodiode sensor (Thorlabs, Newton, NJ, USA) was used to measure light power before each experiment, and the light dose was 2 J/cm^2^. Each experiment included the following groups: cells not treated with PS and not exposed to light (untreated control), cells exposed only to light, cells exposed to the PS without irradiation (to determine dark toxicity), and cells treated with PS and exposed to light (to determine light toxicity). The cells were incubated for 24 h after irradiation at standard cell culture conditions, and the MTT assay was performed according to the previously published protocol [14,41]. The absorbance was measured at a wavelength of 570 nm using a plate reader (Biotek Instruments, Elx-800, Winooski, VT, USA).

For hypoxic conditions, the cells were transferred to the hypoxia station 24 h before adding the compounds. All experimental procedures, including treatment and irradiation, were performed under hypoxic conditions.

To determine the IC_50_ values, the A2780 and MDA-MB-231 cells were seeded at a density of 15 × 10^3^ cells on 96-well plates and incubated overnight at standard cell culture conditions. Cells were treated with compound **1** at concentrations of 100 nM, 200 nM, 500 nM, 600 nM, 800 nM, and 1000 nM and compounds **2a** and **2b** at a concentration range of 0.5 nM, 1 nM, 5 nM, 10 nM, 20 nM, and 50 nM for 24 h. Similar to preliminary experiments, DMSO was used as a control, and the concentration in the cell culture medium did not exceed 0.1%. For hypoxic conditions, the tested concentration range was 100 nM, 200 nM, 500 nM, 600 nM, 800 nM, and 1000 nM for all compounds. The irradiation and MTT assays were performed as a preliminary study.

The IC_50_ values were calculated using the GraphPad Prism 8 software (GraphPad Software, Inc., La Jolla, CA, USA). The variable slope (four parameters) analysis was used to calculate dose and response curves and determine IC_50_ values. To determine IC_50_ values, three biological replications were performed for each compound.

### 2.6. Statistical Analysis

Statistical analysis was performed using GraphPad Prism^®^ 8 (GraphPad Software, Inc., La Jolla, CA, USA). One-way ANOVA followed by Dunnett’s post hoc test was used to determine significance; *p* < 0.05 was considered significant.

## 3. Results and Discussion

### 3.1. Synthesis and Characterization

**Scheme 1:**
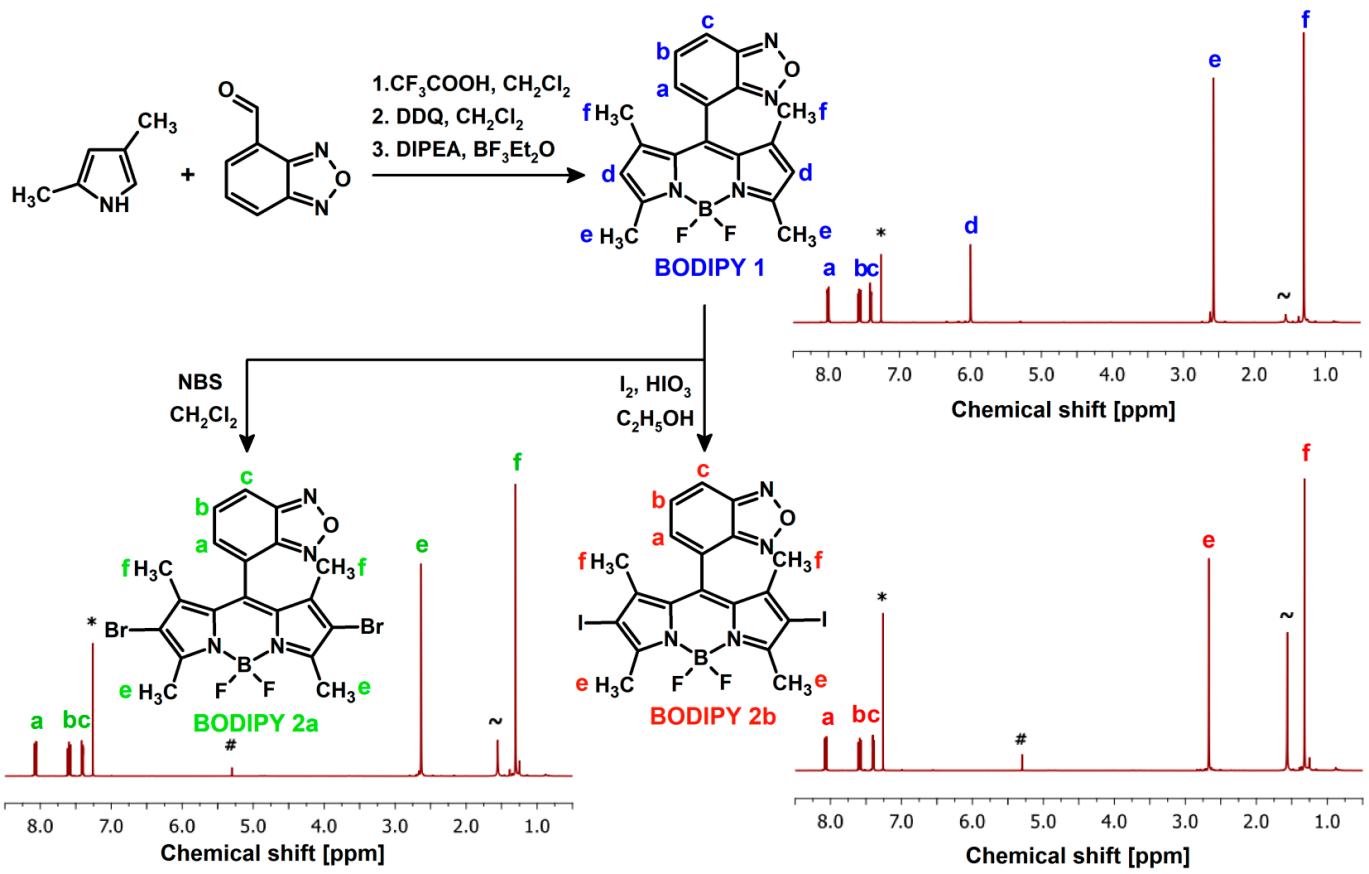
Synthesis of BODIPYs **1**, **2a**, and **2b** with their ^1^H NMR spectra in CDCl_3_. The letters a-f assign NMR signals to the hydrogen atoms in the BODIPY structures. The residual peaks of chloroform, dichloromethane, and water are indicated by symbols *, #, and ~, respectively.

Novel BODIPY **1** possessing a *meso*-benzofurazan substituent was synthesized from 2,4-dimethylpyrrole and 4-formyl-2,1,3-benzoxadiazole in a three-step reaction using trifluoroacetic acid, 2,3-dichloro-5,6-dicyano-1,4-benzoquinone (DDQ), N,N-diisopropylethylamine (DIPEA), and boron trifluoride etherate in dichloromethane following similar procedures for the synthesis of phthalimide-substituted BODIPYs [42,43]. In the next steps, BODIPY **1** was brominated in positions 2 and 6 of the BODIPY core using N-bromosuccinimide (NBS) to give derivative **2a** and then iodinated with the mixture of I_2_ and HIO_3_, thereby resulting in the synthesis of compound **2b**, similar to the previously described procedure [44] (Scheme 1).

Novel compounds **1**, **2a**, and **2b** were characterized using high-resolution mass spectrometry (HRMS), UV-Vis spectrophotometry, and various NMR techniques. The ^1^H NMR spectrum of BODIPY **1** showed six signals (Scheme 1). The signals at 7.41, 7.56, and 8.01 ppm are derived from the phenyl group of a benzoxadiazole substituent at the BODIPY *meso* position. The singlets at 1.30 and 2.57 ppm belong to protons of methyl groups at positions 1 and 7 of the BODIPY core. Another singlet at 6.00 can be assigned to the hydrogen atoms at positions 2 and 6 of the BODIPY core. The NMR spectra of BODIPYs **2a** and **2b** differ from the spectrum of BODIPY **1** due to the lack of the signal at 6.00 ppm, which confirms the introduction of bromine and iodine atoms at positions 2 and 6 of the BODIPY core (Scheme 1). A detailed analysis of the NMR spectra can be found in Supplementary Information (Figures S1–S9, Tables S1–S3). 

### 3.2. Absorption and Emission Properties

**Figure 2:**
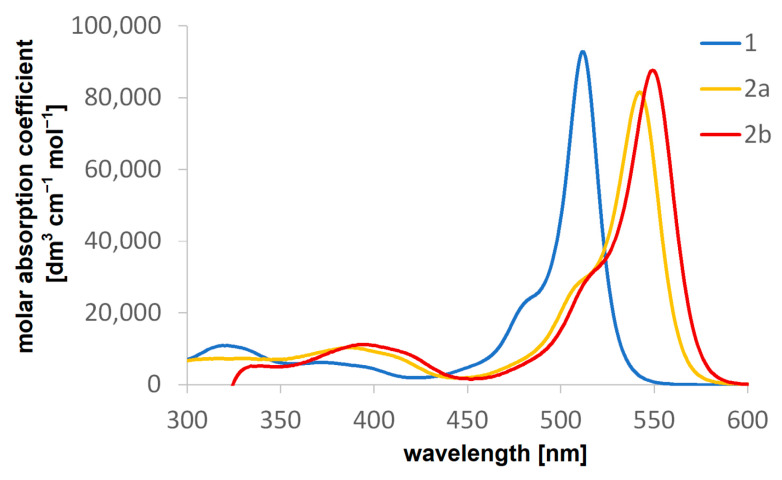
Absorption spectra of compounds **1, 2a**, and **2b** in dichloromethane.

The absorption properties of BODIPYs **1**, **2a**, and **2b** were established in various solvents, including dichloromethane, methanol, ethanol, and dimethyl sulfoxide. Figure 2 presents the UV-Vis absorption spectra of the studied dyes in dichloromethane (DCM), while Figure S13 of Supplementary Information includes the spectra in other solvents.

Table 1 contains the absorption maxima (λ_Abs_) with logarithms of molar absorption coefficients (log ε) in dichloromethane, whereas Table S4 of Supplementary Information presents data for other solvents. The UV-Vis spectra revealed intensive bands in the 450–600 nm range for halogenated BODIPYs **2a** and **2b** and between 425 and 550 nm for BODIPY **1**. These bands can be assigned to the S_0_→S_1_ transition. The lower intensity bands corresponding to the S_0_→S_2_ transition were found between 300 and 425 nm for BODIPY **1** and 350–450 nm for BODIPYs **2a** and **2b**. Data from the literature suggested that *meso*-aryl substitutions in the BODIPY core did not remarkably alter the absorbance and fluorescence spectra (Figure 1). In this study, we also showed that benzofurazan moiety at the *meso* position slightly affected the localization of the absorption maxima, causing only about a 10 nm shift towards longer wavelengths compared with *meso*-phenyl substituted analogs [14,15]. In addition, the bathochromic shift in absorption bands for compounds **2a** (λ_Abs in DCM_ = 542 nm) and **2b** (λ_Abs in DCM_ = 549 nm), in comparison to BODIPY **1** (λ_Abs in DCM_ = 512 nm), results from the presence of iodine and bromine atoms in the BODIPY core. Notably, all studied compounds revealed high log ε values from 4.67 to 4.99 (Table 1 and Table S4 of Supplementary Information).

**Figure 3:**
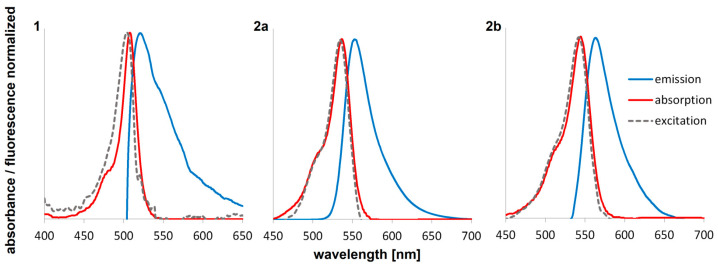
Long-wavelength absorption, excitation, and emission bands for BODIPYs **1**, **2a**, and **2b** in methanol.

The emission spectra of benzofurazan BODIPYs were recorded in methanol (Figure 3), and the obtained fluorescence data, including emission maxima (λ_em_), Stokes shifts (Δλ), and fluorescence quantum yields (Φ_F_), are summarized in Table 1. Regarding the fluorescence quantum yields, all compounds exhibited low Φ_F_ values, with the lowest value calculated for compound **1**. Bumagina et al. suggested that *meso*-aryl substitutions can decrease fluorescence quantum yields [32]. Our data indicate that a *meso*-aryl substitution with benzofurazan moiety may further reduce fluorescence quantum yields.

The tested BODIPYs revealed a weak fluorescence, with the emission maxima at 520, 552, and 563 nm for BODIPYs **1**, **2a**, and **2b**, respectively. The fluorescence quenching by oxadiazole moiety in a benzofurazan substituent is responsible for the minimal fluorescence observed for unsubstituted derivative **1** [30,45]. In contrast, the *meso*-phenyl-substituted BODIPYs are known for their high fluorescence [14,15,46]. However, the benzofurazan-derived fluorescence quenching effect was reduced by heavy atoms, as BODIPYs **2a** and **2b** revealed a higher fluorescence intensity than the unsubstituted analog **1**. Also, the lower fluorescence intensity of iodinated dye **2b** than brominated **2a** agrees with the heavy atom effect caused by the presence of these atoms in positions 2 and 6 of the BODIPY core [6,44,47,48].

### 3.3. Singlet Oxygen Generation Measurements

**Figure 4:**
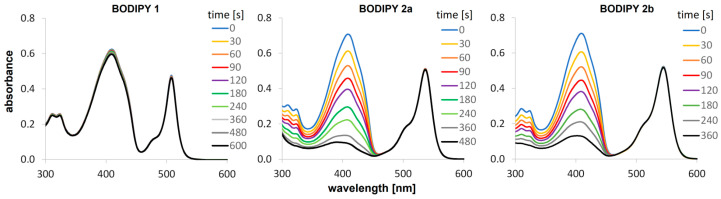
The time-dependent UV-Vis spectra for 1,3-diphenylisobenzofuran (DPBF) upon irradiation at a wavelength of 520 nm with BODIPYs **1**, **2a**, and **2b** in methanol.

Singlet oxygen quantum yields (Φ_Δ_) were determined using a relative method, where Rose Bengal was used as a photosensitizer standard and 1,3-diphenylisobenzofuran (DPBF) as a singlet oxygen quencher. The solutions of compounds **1**, **2a**, **2b**, or Rose Bengal with DPBF were prepared in methanol and irradiated with 520 nm green light. During irradiation, DPBF decomposes and oxidizes, which is observed as decreasing absorption at a wavelength of about 410 nm. The changes in the UV-Vis spectra during measurements are shown in Figure 4, whereas first-order plots of DPBF degradation by singlet oxygen are presented in Figure S14 of Supplementary Information. The calculated values of Φ_Δ_ are presented in Table 1. Compound **1** showed a low ability to generate singlet oxygen, with the value of Φ_Δ_ equal to 0.02, compared to the value obtained for the standard substance, Rose Bengal (Φ_Δ_ = 0.76 [40]). On the other hand, derivatives **2a** and **2b** showed a much higher singlet oxygen generation efficiency with the values of Φ_Δ_ = 0.55 and 0.63. The significantly higher ability to generate singlet oxygen by the compound containing iodine or bromine atoms in positions 2 and 6 of the BODIPY ring is associated with the heavy atom effect. The presence of iodine or bromine atoms at these positions causes the light-excited molecule to transition from the singlet state to the triplet state, where the molecule can react efficiently with molecular oxygen, forming singlet oxygen with high yields [44,49,50].

### 3.4. Cell Viability Evaluation Using the MTT Assay

**Figure 5:**
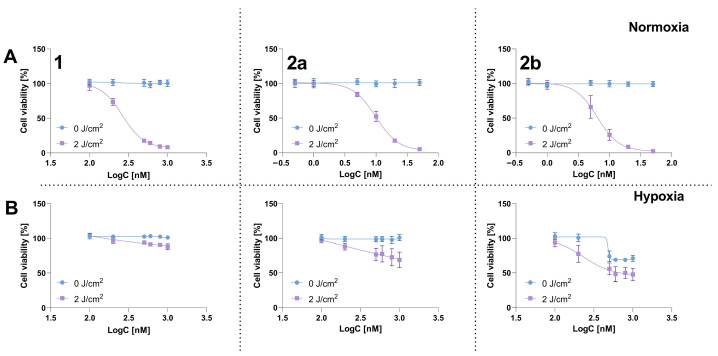
The dose–response curves for compounds **1**, **2a**, and **2b** against MDA-MB-231 cells. Cell viability was measured using the MTT assay 24 h after irradiation (phototoxicity) or without irradiation (dark toxicity) under normoxic (**Panel A**) and hypoxic (**Panel B**) conditions. Data are presented as mean values ± SD, calculated from three independent experiments (except for compound **1** under hypoxic conditions, which was repeated once due to its lack of cytotoxic effect).

**Figure 6:**
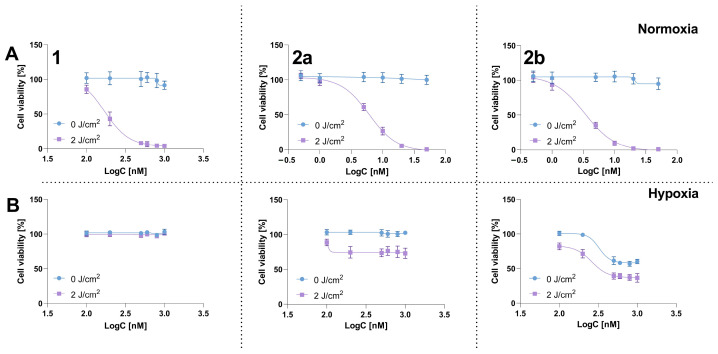
The cytotoxic activity of compounds **1**, **2a**, and **2b** towards A2780 cells. (**Panel A**) presents the results under normoxic conditions, while (**Panel B**) presents the results under hypoxic conditions. Cell viability was measured using the MTT assay 24 h after irradiation (phototoxicity) and without exposure to light (dark toxicity). Data are presented as mean values ± SD, calculated from three independent experiments (except for compound **1** under hypoxic conditions, which was repeated once due to its lack of cytotoxic effect).

Such features of photosensitizers from the BODIPY family, including stability under various environmental conditions, high molar extinction coefficients, and resistance to photobleaching, make these compounds excellent candidates for PDT applications [6]. Moreover, the BODIPY core is a versatile scaffold that can be modified to improve photophysical and photochemical properties, thereby enhancing biological activity [6,32]. This assertion is supported by several in vitro studies in which BODIPYs demonstrated excellent phototoxic activity, with IC_50_ values in the nanomolar range [14,51,52]. As presented in Figure 5 and Figure 6, BODIPYs **1**, **2a**, and **2b** exerted cytotoxic activity towards the A2780 and MDA-MB-231 cell lines after irradiation and did not affect cell viability without light exposure. The results of the preliminary experiment used to determine the concentration range for IC_50_ value determination are presented in Figure S15. The morphology of the A2780 and MDA-MB-231 cells after treatment with BODIPYs **1**, **2a**, and **2b** is shown in Figures S16–S21. The statistical analysis and significance of the obtained results are presented in Supplementary Materials (Figures S22–S25).

The most potent activity was observed for compound **2b**, with IC_50_ values of 3.55 ± 0.44 nM and 6.47 ± 1.73 nM for the A2780 and MDA-MB-231 cells, respectively (Table 2).

Thus, our studies confirmed that the presence of iodine atoms at positions 2 and 6 can enhance the cytotoxic activity, probably due to the higher singlet oxygen generation. The insertion of bromine at positions 2 and 6 also ensures prominent cytotoxic activity, with IC_50_ values of 6.08 ± 0.56 nM and 10.26 ± 1.06 nM for the A2780 and MDA-MB-231 cells (Table 2). The absence of a heavy atom in compound **1** resulted in a lower activity compared to compounds **2a** and **2b**; however, the IC_50_ values for both cell lines were in the nanomolar concentration range. Badon and co-workers also observed a similar effect for a diaminophenyl-functionalized cationic BODIPY [53]. The authors demonstrated that the iodinated BODIPY exhibited a higher singlet oxygen quantum yield (0.55) than its brominated counterpart (0.13). The authors also found that the iodinated and brominated derivatives showed potent cytotoxic activity against human cervical adenocarcinoma (HeLa) and human breast adenocarcinoma (MCF-7) cells. The iodinated derivative showed IC_50_ values of 64 nM and 48.57 nM for HeLa and MCF-7 cells, respectively, while the brominated derivative exhibited IC_50_ values of 59.27 nM and 49.35 nM for HeLa and MCF-7 cells, respectively. On the other hand, non-halogenated counterparts did not affect cell viability [53]. In our studies, the non-halogenated derivative **1** decreased the cell viability with IC_50_ values of 182.72 ± 25.81 and 279.34 ± 10.30 nM for the A2780 and MDA-MB-231 cells, respectively, despite the singlet oxygen quantum yield reaching a value of 0.02 (approximately 30 times lower than that of **2b**). Interestingly, there is also approximately a 30-fold difference in the IC_50_ values between compounds **1** and **2b**.

Since hypoxia is one of the well-known factors that can limit PDT effectiveness, we tested all compounds under hypoxic conditions. Compound **1** was inactive under hypoxic conditions in both cell lines in the concentration range corresponding to the normoxic conditions (Figure 5 and Figure 6). The results obtained under normoxic conditions exhibited the same trends as those under hypoxic conditions. The most potent cytotoxic effect was observed for compound **2b** with IC_50_ values of 293.77 ± 66.76 nM and 429.00 ± 11.72 nM for A2780 and MDA-MB-231 cells (Table 3). However, it should be noted that under hypoxic conditions at the tested concentration range, compound **2b** also affects cell viability without irradiation. At concentrations of 1000 nM, 800 nM, 600 nM, and 500 nM, compound **2b** decreased cell viability to ~60% in A2780 cells (Figure 6, Table S5). Compound **2b** demonstrated a more potent effect without irradiation on ovarian cancer cells under hypoxic conditions since, for MDA-MB-231 cells, this compound decreased cell viability to ~70% compared to the control cells (Figure 5 and Figure 6, Table S5). Compound **2a** under hypoxic conditions reduced cell viability after irradiation at the highest dose to ~70% for both cell lines (Table S6), while it did not affect cell viability without irradiation (Table S5). Therefore, it can be concluded that bromine substitution led to a less photocytoxic effect but also exerted a less cytotoxic effect. Although the generation of singlet oxygen is lower for BODIPYs **2a** and **2b** (0.55 and 0.63, respectively), compared to the already described N,N-dimethylaminopropoxyphenyl derivative in the literature, both compounds also demonstrated activity under hypoxic conditions. In contrast, the BODIPY with the N,N-dimethylaminopropoxyphenyl group at the *meso* position only exerted cytotoxic activity towards the human prostate carcinoma (LNCaP) cell line under normoxic conditions and was inactive in hypoxia [14]. Therefore, considering singlet oxygen generation, the phototoxic activity of compounds **2a** and **2b** could be related to both type I and type II photodynamic reactions in their phototoxic mechanism of action. However, further studies are needed to verify this hypothesis.

## 4. Conclusions

A simple procedure for synthesizing novel boron dipyrromethene derivatives with a heterocyclic, benzoxadiazole substituent at the *meso* position was elaborated. Obtained dyes were characterized using mass spectrometry and various NMR techniques and subjected to photochemical and cytotoxicity studies. The presence of benzoxadiazole moiety only slightly affected the localization of the absorption maxima but resulted in fluorescence quenching compared with *meso*-phenyl-substituted analogs. In addition, brominated and iodinated analogs revealed a high ability to generate singlet oxygen. The in vitro studies using human ovarian carcinoma (A2780) and human breast adenocarcinoma (MDA-MB-231) cells revealed high light-induced cytotoxicity of BODIPYs containing heavy atoms with very low IC_50_ values in the 3.5–10.3 nM range. Further studies revealed that both compounds demonstrated phototoxic activity also under hypoxic conditions. The most potent cytotoxic effect in these conditions was observed for the iodinated BODIPY analog with IC_50_ values of about 0.3 and 0.4 μM for the A2780 and MDA-MB-231 cells, respectively. However, it should be noted that this compound also affects cell viability without irradiation under hypoxic conditions at the tested concentration range. The brominated analog showed less phototoxic effect but also exerted less cytotoxic effect itself. Nevertheless, both BODIPYs with heavy atoms are promising candidates for further research. The results of this study highlighted the advantages and some potential drawbacks of BODIPY compounds with heavy atoms and benzoxadiazole moiety as a useful scaffold in medicinal chemistry for designing new photosensitizers. However, more detailed studies are required to fully characterize the potential of the tested compounds, including mechanistic and functional analyses to elucidate their molecular mechanisms of action. This knowledge could also help identify and overcome potential resistance mechanisms, particularly under hypoxic conditions. Moreover, preclinical in vivo studies could further define the applicability of the tested BODIPYs.

## Figures and Tables

**Table 1:** UV-Vis absorption maxima (λ_Abs_), logarithms of molar absorption coefficients (log ε), emission wavelengths (λ_em_), Stokes shifts (Δλ), quantum yields of fluorescence (Φ_F_), and singlet oxygen generation yields (Φ_Δ_) of BODIPYs **1**, **2a** and **2b** in methanol.

Compound	λ_Abs_ [nm]	log ε ± SD	λ_em_ [nm]	Δλ [nm]	Φ_F_ ± SD	Φ_Δ_ ± SD
**1**	512	4.98 ± 0.04	520	8	0.002 ± 0.001	0.02 ± 0.01
**2a**	542	4.91 ± 0.03	552	10	0.097 ± 0.004	0.55 ± 0.01
**2b**	549	4.94 ± 0.05	563	14	0.010 ± 0.001	0.63 ± 0.01

**Table 2:** The IC_50_ values for tested compounds under normoxic conditions against A2780 and MDA-MB-231 cells. Data presented as mean ± SD from three independent experiments.

	IC_50_ [nM]			
	A2780		MDA-MB-231	
	0 J/cm^2^	2 J/cm^2^	0 J/cm^2^	2 J/cm^2^
**1**	>1000	182.72 ± 25.81	>1000	279.34 ± 10.30
**2a**	>50	6.08 ± 0.56	>50	10.26 ± 1.06
**2b**	>50	3.55 ± 0.44	>50	6.47 ± 1.73

**Table 3:** The IC_50_ values for tested compounds under hypoxic conditions against A2780 and MDA-MB-231 cells. Data presented as mean ± SD from at least two independent experiments.

	IC_50_ [nM]			
	A2780		MDA-MB-231	
	0 J/cm^2^	2 J/cm^2^	0 J/cm^2^	2 J/cm^2^
**1**	>1000	>1000	>1000	>1000
**2a**	>1000	>1000	>1000	>1000
**2b**	>1000	293.77 ± 66.76	>1000	429.00 ± 11.72

## Data Availability

Data are contained within the article/Supplementary Material, further inquiries can be directed to the corresponding authors.

## Supplementary Materials

The following supporting information can be downloaded at https://www.mdpi.com/article/10.3390/biomedicines13020297/s1, Figure S1: ^1^H NMR of BODIPY **1** in deuterated chloroform; Figure S2: ^13^C NMR of BODIPY **1**; Figure S3: ^1^H and (^13^C) chemical shift values [ppm] and key correlations observed in NMR spectra of BODIPY **1**; Figure S4: ^1^H NMR of BODIPY **2a** in deuterated chloroform; Figure S5: ^13^C NMR of BODIPY **2a**; Figure S6: ^1^H and (^13^C) chemical shift values [ppm] and key correlations observed in NMR spectra of **2b**; Figure S7: ^1^H NMR of BODIPY **2b** in deuterated chloroform; Figure S8: ^13^C NMR of BODIPY **2b**; Figure S9: ^1^H and (^13^C) chemical shift values [ppm] and key correlations observed in NMR spectra of **2b**; Figure S10: HRMS of BODIPY **1**; Figure S11: HRMS of BODIPY **2a**; Figure S12: HRMS of BODIPY **2b**; Figure S13: Absorption spectra of compounds **1**, **2a**, and **2b**; Figure S14: The first-order plots for the oxidation of the DPBF for BODIPYs **1**, **2a**, and **2b** in methanol; Figure S15: The preliminary results for compounds **1**, **2a**, and **2b** performed in normoxic conditions towards MDA-MB-231 cells; Figure S16: The A2780 morphology after treatment with BODIPY **1**; Figure S17: The A2780 cells morphology after treatment with BODIPY **2a**; Figure S18: The A2780 morphology after treatment with BODIPY **2b**; Figure S19: The MDA-MB-231 cells morphology after treatment with BODIPY 1; Figure S20: The MDA-MB-231 cells morphology after treatment with BODIPY **2a**; Figure S21: The MDA-MB-231 cells morphology after treatment with BODIPY **2b**; Figure S22. The viability of MDA-MB-231 cells after treatment with BODIPYs **1**, **2a**, and **2b** under normoxic conditions; Figure S23. The viability of MDA-MB-231 cells after treatment with BODIPYs **1**, **2a**, and **2b** under hypoxic conditions; Figure S24. The viability of A2780 cells after treatment with BODIPYs **1**, **2a**, and **2b** under normoxic conditions; Figure S25. The viability of A2780 cells after treatment with BODIPYs **1**, **2a**, and **2b** under hypoxic conditions; Table S1: NMR data for compound **1**, including key correlations determined from ^1^H-^1^H COSY, ^1^H-^13^C HSQC, and ^1^H-^13^C HMBC spectra; Table S2: NMR data for BODIPY **2a**, including key correlations determined from ^1^H-^1^H COSY, ^1^H-^13^C HSQC, and ^1^H-^13^C HMBC spectra; Table S3: NMR data for **2b**, including key correlations determined from ^1^H-^1^H COSY, ^1^H-^13^C HSQC, and ^1^H-^13^C HMBC spectra; Table S4: UV-Vis absorption maxima (λ_Abs_) and logarithms of molar absorption coefficients (log ε) of compounds **1**, **2a**, and **2b** in various solvents; Table S5: The cell viability of A2780 and MDA-MB-231 treated with **2a** and **2b** under hypoxic conditions without exposure to light; Table S6: The cell viability of A2780 and MDA-MB-231 treated with **2a** and **2b** under hypoxic conditions after irradiation at a light dose of 2 J/cm^2^.

## References

[1] Zhang D., Liu L., Zhang X., Lu J., Jiang X.-D. (2024). Rational Design of Photofunctional Dyes BODIPYs/Aza-BODIPYs and Applications for Photocatalysis, Photoelectric Conversion and Thermochromic Materials. Resour. Chem. Mater..

[2] Ilhan H., Cakmak Y. (2023). Functionalization of BODIPY Dyes with Additional C–N Double Bonds and Their Applications. Top. Curr. Chem..

[3] Das S., Dey S., Patra S., Bera A., Ghosh T., Prasad B., Sayala K.D., Maji K., Bedi A., Debnath S. (2023). BODIPY-Based Molecules for Biomedical Applications. Biomolecules.

[4] Zhang S., Qu Y., Zhang D., Li S., Tang F., Ding A., Hu L., Zhang J., Wang H., Huang K. (2024). Rational Design and Biological Application of Hybrid Fluorophores. Chem. Eur. J..

[5] Cheng H., Cao X., Zhang S., Zhang K., Cheng Y., Wang J., Zhao J., Zhou L., Liang X., Yoon J. (2023). BODIPY as a Multifunctional Theranostic Reagent in Biomedicine: Self-Assembly, Properties, and Applications. Adv. Mater..

[6] Malacarne M.C., Gariboldi M.B., Caruso E. (2022). BODIPYs in PDT: A Journey through the Most Interesting Molecules Produced in the Last 10 Years. Int. J. Mol. Sci..

[7] Yadav I.S., Misra R. (2023). Design, Synthesis and Functionalization of BODIPY Dyes: Applications in Dye-Sensitized Solar Cells (DSSCs) and Photodynamic Therapy (PDT). J. Mater. Chem. C.

[8] Lo P., Ng D.K.P., Pandey R.K., Zimcik P. (2023). Photodynamic Therapy: An Innovative and Versatile Treatment Modality Triggered by Light. ChemPlusChem.

[9] Michalak M., Mazurkiewicz S., Szymczyk J., Ziental D., Sobotta Ł. (2023). Photodynamic Therapy Applications—Review. JMS.

[10] Hong L., Li J., Luo Y., Guo T., Zhang C., Ou S., Long Y., Hu Z. (2022). Recent Advances in Strategies for Addressing Hypoxia in Tumor Photodynamic Therapy. Biomolecules.

[11] Udomsak W., Kucinska M., Pospieszna J., Dams-Kozlowska H., Chatuphonprasert W., Murias M. (2024). Antioxidant Enzymes in Cancer Cells: Their Role in Photodynamic Therapy Resistance and Potential as Targets for Improved Treatment Outcomes. Int. J. Mol. Sci..

[12] Liu M., Ma S., She M., Chen J., Wang Z., Liu P., Zhang S., Li J. (2019). Structural Modification of BODIPY: Improve Its Applicability. Chin. Chem. Lett..

[13] Kamkaew A., Lim S.H., Lee H.B., Kiew L.V., Chung L.Y., Burgess K. (2013). BODIPY Dyes in Photodynamic Therapy. Chem. Soc. Rev..

[14] Piskorz J., Porolnik W., Kucinska M., Dlugaszewska J., Murias M., Mielcarek J. (2021). BODIPY-Based Photosensitizers as Potential Anticancer and Antibacterial Agents: Role of the Positive Charge and the Heavy Atom Effect. ChemMedChem.

[15] Loudet A., Burgess K. (2007). BODIPY Dyes and Their Derivatives: Syntheses and Spectroscopic Properties. Chem. Rev..

[16] Prieto-Montero R., Prieto-Castañeda A., Katsumiti A., Sola-Llano R., Agarrabeitia A.R., Cajaraville M.P., Ortiz M.J., Martinez-Martinez V. (2022). Red haloBODIPYs as Theragnostic Agents: The Role of the Substitution at *Meso* Position. Dye. Pigment..

[17] Hu W., Zhang X.-F., Lu X., Lan S., Tian D., Li T., Wang L., Zhao S., Feng M., Zhang J. (2018). Modifying the Meso -Phenyl with Electron Donating Amino Groups Strongly Enhances BODIPY’s Ability as Good Singlet Oxygen Photosensitizer. Dye. Pigment..

[18] Ko S., Kim C.Y., Damodar K., Lim H.M., Kim J.H., Lee C.-H., Lee J.T. (2018). Substituents Modification of *Meso*-Aryl BODIPYs for Tuning Photophysical Properties. Tetrahedron.

[19] Guseva G.B., Antina E.V., Berezin M.B., Pavelyev R.S., Kayumov A.R., Sharafutdinov I.S., Lisovskaya S.A., Lodochnikova O.A., Islamov D.R., Usachev K.S. (2020). *Meso*-Substituted-BODIPY Based Fluorescent Biomarker: Spectral Characteristics, Photostability and Possibilities for Practical Application. J. Photochem. Photobiol. A Chem..

[20] Hu W., Zhang X.-F., Lu X., Lan S., Tian D., Li T., Wang L., Zhao S., Feng M., Zhang J. (2018). Attaching Electron Donating Groups on the *Meso*-Phenyl and *Meso*-Naphthyl Make Aryl Substituted BODIPYs Act as Good Photosensitizer for Singlet Oxygen Formation. J. Lumin..

[21] Shi W.-J., Chen R., Yang J., Wei Y.-F., Guo Y., Wang Z.-Z., Yan J., Niu L. (2022). Novel *Meso*-Benzothiazole-Substituted BODIPY-Based AIE Fluorescent Rotor for Imaging Lysosomal Viscosity and Monitoring Autophagy. Anal. Chem..

[22] Liu R., Qian Y. (2024). NIR Ditriphenylamine Indole-BODIPY Photosensitizer: Synthesis, Photodynamic Therapy in A549 Cells and Two-Photon Fluorescence Imaging in Zebrafish. Spectrochim. Acta Part A Mol. Biomol. Spectrosc..

[23] Gonçalves R.C.R., Teixeira F., Peñalver P., Costa S.P.G., Morales J.C., Raposo M.M.M. (2024). Designing Antitrypanosomal and Antileishmanial BODIPY Derivatives: A Computational and In Vitro Assessment. Molecules.

[24] Tursynova N., Helena Maliszewska I., Jóźwiak K., Sokolnicki J., Kochel A., Lipkowski P., Bartkiewicz S., Filarowski A. (2024). The Photoinactivation of Pathogenic Bacteria Using Synthesized Benzodioxole-BODIPY Dyes. J. Photochem. Photobiol. A Chem..

[25] Mancini R.S., Barden C.J., Weaver D.F., Reed M.A. (2021). Furazans in Medicinal Chemistry. J. Med. Chem..

[26] Verdoliva V., Digilio G., Saviano M., De Luca S. (2021). Thio-Conjugation of Substituted Benzofurazans to Peptides: Molecular Sieves Catalyze Nucleophilic Attack on Unsaturated Fused Rings. Catal. Sci. Technol..

[27] Li L., Kracht J., Peng S., Bernhardt G., Buschauer A. (2003). Synthesis and Pharmacological Activity of Fluorescent Histamine H1 Receptor Antagonists Related to Mepyramine. Bioorg. Med. Chem. Lett..

[28] Kang J., Huo F., Ning P., Meng X., Chao J., Yin C. (2017). Two Red-Emission Single and Double ‘Arms’ Fluorescent Materials Stemed from ‘One-Pot’ Reaction for Hydrogen Sulfide Vivo Imaging. Sens. Actuators B Chem..

[29] Ding X., Wang Q., Chen D., Chen Y., Pan W., Sun Q., Chen Q., Han X. (2023). A Fluorescent Probe Based on BODIPY for Hydrogen Sulfide Imaging in Living Cells and Zebrafish. Adv. Agrochem..

[30] Xiang H.-J., Tham H.P., Nguyen M.D., Fiona Phua S.Z., Lim W.Q., Liu J.-G., Zhao Y. (2017). An Aza-BODIPY Based near-Infrared Fluorescent Probe for Sensitive Discrimination of Cysteine/Homocysteine and Glutathione in Living Cells. Chem. Commun..

[31] Parisi C., Longobardi G., Graziano A.C.E., Fraix A., Conte C., Quaglia F., Sortino S. (2022). A Molecular Dyad Delivered by Biodegradable Polymeric Nanoparticles for Combined PDT and NO-PDT in Cancer Cells. Bioorg. Chem..

[32] Bumagina N.A., Antina E.V., Ksenofontov A.A., Antina L.A., Kalyagin A.A., Berezin M.B. (2022). Basic Structural Modifications for Improving the Practical Properties of BODIPY. Coord. Chem. Rev..

[33] Magde D., Wong R., Seybold P.G. (2002). Fluorescence Quantum Yields and Their Relation to Lifetimes of Rhodamine 6G and Fluorescein in Nine Solvents: Improved Absolute Standards for Quantum Yields. Photochem. Photobiol..

[34] Lim S.H., Thivierge C., Nowak-Sliwinska P., Han J., van den Bergh H., Wagnières G., Burgess K., Lee H.B. (2010). In Vitro and In Vivo Photocytotoxicity of Boron Dipyrromethene Derivatives for Photodynamic Therapy. J. Med. Chem..

[35] Porolnik W., Kasprzycka M., Podciechowska K., Teubert A., Piskorz J. (2022). Synthesis and Spectroscopic Properties of Novel Dipyrrole and Tetrapyrrole-Based Photosensitizers with Various Biphenylyl Substituents. Tetrahedron.

[36] Kryjewski M., Rebis T., Milczarek G., Gdaniec Z., Goslinski T., Mielcarek J. (2016). Magnesium(ii) 1-(1-Adamantylsulfanyl)Phthalocyanine—Synthesis, Photochemical and Electrochemical Properties. New J. Chem..

[37] Karstens T., Kobs K. (1980). Rhodamine B and Rhodamine 101 as Reference Substances for Fluorescence Quantum Yield Measurements. J. Phys. Chem..

[38] Porolnik W., Ratajczak M., Mackowiak A., Murias M., Kucinska M., Piskorz J. (2024). Liposomal Formulations of Novel BODIPY Dimers as Promising Photosensitizers for Antibacterial and Anticancer Treatment. Molecules.

[39] Lagorio M.G., Dicelio L.E., San Roman E.A., Braslavsky S.E. (1989). Quantum Yield of Singlet Molecular Oxygen Sensitization by Copper(II) Tetracarboxyphthalocyanine. J. Photochem. Photobiol. B Biol..

[40] Kochevar I.E., Redmond R.W. (2000). [2] Photosensitized Production of Singlet Oxygen. Methods in Enzymology.

[41] Kucinska M., Skupin-Mrugalska P., Szczolko W., Sobotta L., Sciepura M., Tykarska E., Wierzchowski M., Teubert A., Fedoruk-Wyszomirska A., Wyszko E. (2015). Phthalocyanine Derivatives Possessing 2-(Morpholin-4-Yl)Ethoxy Groups as Potential Agents for Photodynamic Therapy. J. Med. Chem..

[42] Piskorz J., Dlugaszewska J., Porolnik W., Teubert A., Mielcarek J. (2020). Boron-Dipyrromethene Derivatives Bearing N-Alkyl Phthalimide and Amine Substituents of Potential Application in the Photoinactivation of Bacteria. Dye. Pigment..

[43] Galeotti F., Calabrese V., Cavazzini M., Quici S., Poleunis C., Yunus S., Bolognesi A. (2010). Self-Functionalizing Polymer Film Surfaces Assisted by Specific Polystyrene End-Tagging. Chem. Mater..

[44] Krzemien W., Rohlickova M., Machacek M., Novakova V., Piskorz J., Zimcik P. (2021). Tuning Photodynamic Properties of BODIPY Dyes, Porphyrins’ Little Sisters. Molecules.

[45] Shen B., Qian Y., Qi Z., Lu C., Sun Q., Xia X., Cui Y. (2017). Near-Infrared BODIPY-Based Two-Photon ClO^−^ Probe Based on Thiosemicarbazide Desulfurization Reaction: Naked-Eye Detection and Mitochondrial Imaging. J. Mater. Chem. B.

[46] Banfi S., Caruso E., Zaza S., Mancini M., Gariboldi M.B., Monti E. (2012). Synthesis and Photodynamic Activity of a Panel of BODIPY Dyes. J. Photochem. Photobiol. B Biol..

[47] Wang J., Gong Q., Wang L., Hao E., Jiao L. (2020). The Main Strategies for Tuning BODIPY Fluorophores into Photosensitizers. J. Porphyr. Phthalocyanines.

[48] May A.K., Chiyumba C., Harris J., Mack J., Nyokong T. (2022). Photodynamic Antimicrobial Activities of Halogenated 3,5-Dimethyl- and 1,3,5,7-Tetramethyl-*Meso*-Pentafluorophenyl BODIPY Dyes. J. Porphyr. Phthalocyanines.

[49] Çınar H.Ş., Özçelik Ş., Kaya K., Kutlu Ö.D., Erdoğmuş A., Gül A. (2020). Synthesis and Photophysical Properties of Monomeric and Dimeric Halogenated Aza-BODIPYs. J. Mol. Struct..

[50] Turksoy A., Yildiz D., Akkaya E.U. (2019). Photosensitization and Controlled Photosensitization with BODIPY Dyes. Coord. Chem. Rev..

[51] Caruso E., Malacarne M.C., Marras E., Papa E., Bertato L., Banfi S., Gariboldi M.B. (2020). New BODIPYs for Photodynamic Therapy (PDT): Synthesis and Activity on Human Cancer Cell Lines. Bioorg. Med. Chem..

[52] Caruso E., Gariboldi M., Sangion A., Gramatica P., Banfi S. (2017). Synthesis, Photodynamic Activity, and Quantitative Structure-Activity Relationship Modelling of a Series of BODIPYs. J. Photochem. Photobiol. B Biol..

[53] Badon I.W., Jee J.-P., Vales T.P., Kim C., Lee S., Yang J., Yang S.K., Kim H.-J. (2023). Cationic BODIPY Photosensitizers for Mitochondrion-Targeted Fluorescence Cell-Imaging and Photodynamic Therapy. Pharmaceutics.

