# Supplementary material for: Novel BODIPY Dyes with a Meso-Benzoxadiazole Substituent: Synthesis, Photophysical Studies, and Cytotoxic Activity Under Normoxic and Hypoxic Conditions
Source: Biomedicines. 2025 Jan 25;13(2):297. doi: 10.3390/biomedicines13020297 (PMC11853430; doi:10.3390/biomedicines13020297)
Supplement: Supplementary file 1 [file biomedicines-13-00297-s001.zip › biomedicines-3412911-supplementary.pdf]

Novel BODIPY Dyes with a *Meso*-Benzoxadiazole Substituent: Synthesis, Photophysical Studies, and Cytotoxic Activity Under Normoxic and Hypoxic Conditions

Weronika Porolnik[1,2,3], Natalia Karpinska[2], Marek Murias[1], Jaroslaw Piskorz[2]*, Malgorzata Kucinska[1]*

[1] Chair and Department of Toxicology, Poznan University of Medical Sciences, 3 Rokietnicka Street, 60-806 Poznan, Poland; w.porolnik@op.pl (W.P.); marek.murias@ump.edu.pl (M.M.)
[2] Chair and Department of Inorganic and Analytical Chemistry, Poznan University of Medical Sciences, 3 Rokietnicka Street, 60-806 Poznan, Poland; nataliakarpinska20@gmail.com
[3] Doctoral School, Poznan University of Medical Sciences, 70 Bukowska Street, 60-812 Poznan, Poland

* Correspondence: piskorzj@ump.edu.pl (J.P.); kucinska@ump.edu.pl (M.K.)

SUPPLEMENTARY INFORMATION

Table of contents

Figure S1. ^{1}H NMR of BODIPY **1** in deuterated chloroform.. 4

Figure S2. ^{13}C NMR of BODIPY **1**. 5

Table S1. NMR data for compound **1,** including key correlations determined from ^{1}H-^{1}H COSY, ^{1}H-^{13}C HSQC, and ^{1}H-^{13}C HMBC spectra. 5

Figure S3. ^{1}H and (^{13}C) chemical shift values [ppm] and key correlations observed in NMR spectra of BODIPY **1**. 6

Figure S4. ^{1}H NMR of BODIPY **2a** in deuterated chloroform...................................... 7

Figure S5. ^{13}C NMR of BODIPY **2a**. 7

Table S2. NMR data for BODIPY **2a,** including key correlations determined from ^{1}H-^{1}H COSY, ^{1}H-^{13}C HSQC, and ^{1}H-^{13}C HMBC spectra. 7

Figure S6. ^{1}H and (^{13}C) chemical shift values [ppm] and key correlations observed in NMR spectra of **2b**. 8

Figure S7. ^{1}H NMR of BODIPY **2b** in deuterated chloroform 9

Figure S8. ^{13}C NMR of BODIPY **2b**. 9

Table S3. NMR data for **2b,** including key correlations determined from ^{1}H-^{1}H COSY, ^{1}H-^{13}C HSQC, and ^{1}H-^{13}C HMBC spectra. 9

Figure S9. ^{1}H and (^{13}C) chemical shift values [ppm] and key correlations observed in NMR spectra of **2b**. 10

Figure S10. HRMS spectra of BODIPY **1**. 12

Figure S11. HRMS spectra of BODIPY **2a**. 14

Figure S12. HRMS spectra of BODIPY **2b**. 16

Figure S13. Absorption spectra of compounds **1, 2a,** and **2b**. 17

Table S4. UV-Vis absorption maxima (λ_{Abs}) and logarithms of molar absorption coefficients (log ε ± SD) of compounds **1, 2a,** and **2b** in various solvents. 18

Figure S14. The first-order plots for the oxidation of the DPBF for BODIPYs **1**, **2a,** and **2b** in methanol. 19

Figure S15. The preliminary results for compounds **1**, **2a**, and **2b** towards MDA-MB-231 cells performed under normoxic conditions (screening experiment). 20

Figure S16. The A2780 morphology after treatment with BODIPY **1**. 20

Figure S17. The A2780 cells morphology after treatment with BODIPY **2a**. 21

Figure S18. The A2780 morphology after treatment with BODIPY **2b**. 22

Figure S19. The MDA-MB-231 cells morphology after treatment with BODIPY **1**. 23

Figure S20. The MDA-MB-231 cells morphology after treatment with BODIPY **2a**. 24

Figure S21. The MDA-MB-231 cells morphology after treatment with BODIPY **2b**. 25

Figure S22. The viability of MDA-MB-231 cells after treatment with BODIPYs **1**, **2a**, and **2b** under normoxic conditions. 26

Figure S23. The viability of MDA-MB-231 cells after treatment with BODIPYs **1**, **2a**, and **2b** under hypoxic conditions. 27

Figure S24. The viability of A2780 cells after treatment with BODIPYs **1**, **2a**, and **2b** under normoxic conditions. 28

Figure S25. The viability of A2780 cells after treatment with BODIPYs **1**, **2a**, and **2b** under hypoxic conditions. 29

Table S5. The cell viability of A2780 and MDA-MB-231 treated with **2a** and **2b** under hypoxic conditions without exposure to light. 30

Table S6. The cell viability of A2780 and MDA-MB-231 treated with **2a** and **2b** under hypoxic conditions after irradiation at a light dose of 2 J/cm^2. 31

NMR data

Compound 1

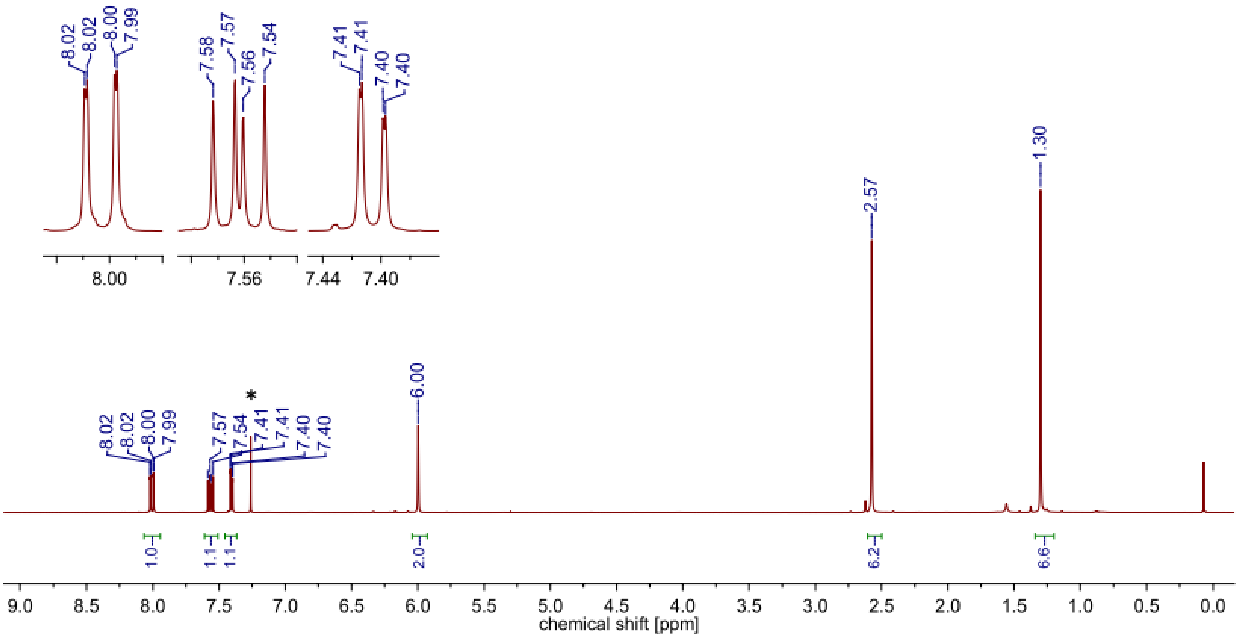

Figure S1. ^{1}H NMR of BODIPY **1** in deuterated chloroform. The symbols * indicate chloroform residual peak.

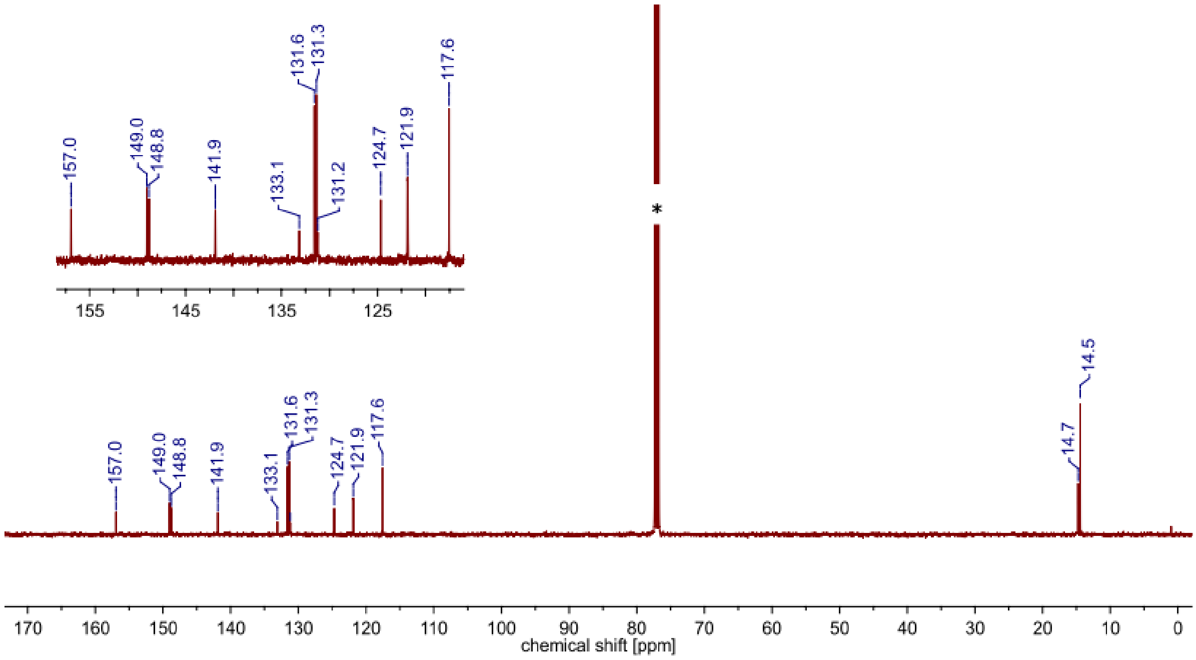

Figure S2. ^{13}C NMR of BODIPY **1**. The symbol * indicates chloroform residual peak.

Table S1. NMR data for compound **1,** including key correlations determined from ^{1}H-^{1}H COSY, ^{1}H-^{13}C HSQC, and ^{1}H-^{13}C HMBC spectra.

δ_H [ppm]	Multiplicity (J_{H-H} w Hz)	^{1}H-^{1}H COSY δ_H [ppm]	^{1}H-^{13}C HSQC δ_C [ppm]	^{1}H-^{13}C HMBC δ_C [ppm]
1.30	s	2.57; 6.00	14.5	121.9; 131.2; 141.9; 157.0
2.57	s	1.30	14.7	121.9; 141.9; 157.0
6.00	s	1.30	121.9	14.5; 131.2; 141.9; 157.0
7.41	dd (6.5; 1)	7.56	131.6	117.6; 131.2; 133.1; 149.0
7.56	dd (9; 7)	7.41; 8.01	131.3	124.7; 149.0
8.01	dd (9; 1)	7.57	117.6	124.7; 131.3; 148.8
Carbon signals [ppm]: 157.0; 149.0; 148.8; 141.9; 133.1; 131.6; 131.3; 131.2; 124.7; 121.9; 117.6; 14.7; 14.5.				

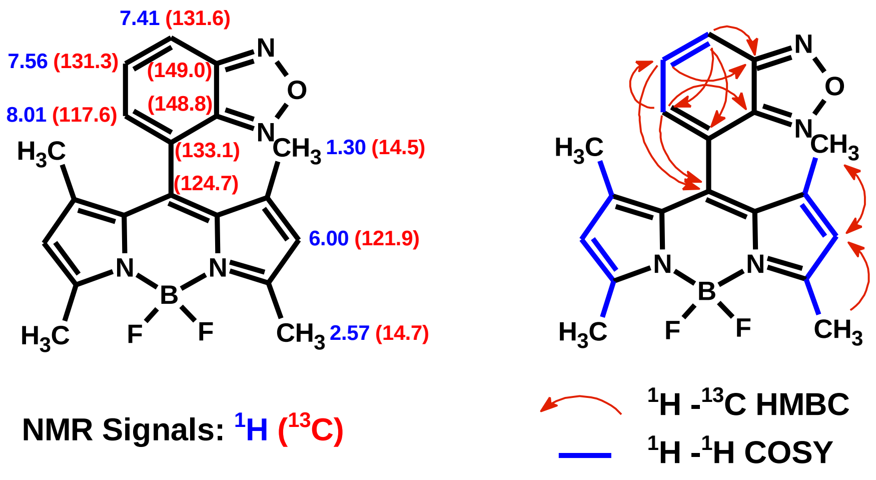

Figure S3. ^{1}H and (^{13}C) chemical shift values [ppm] and key correlations observed in NMR spectra of BODIPY **1**.

Compound 2a

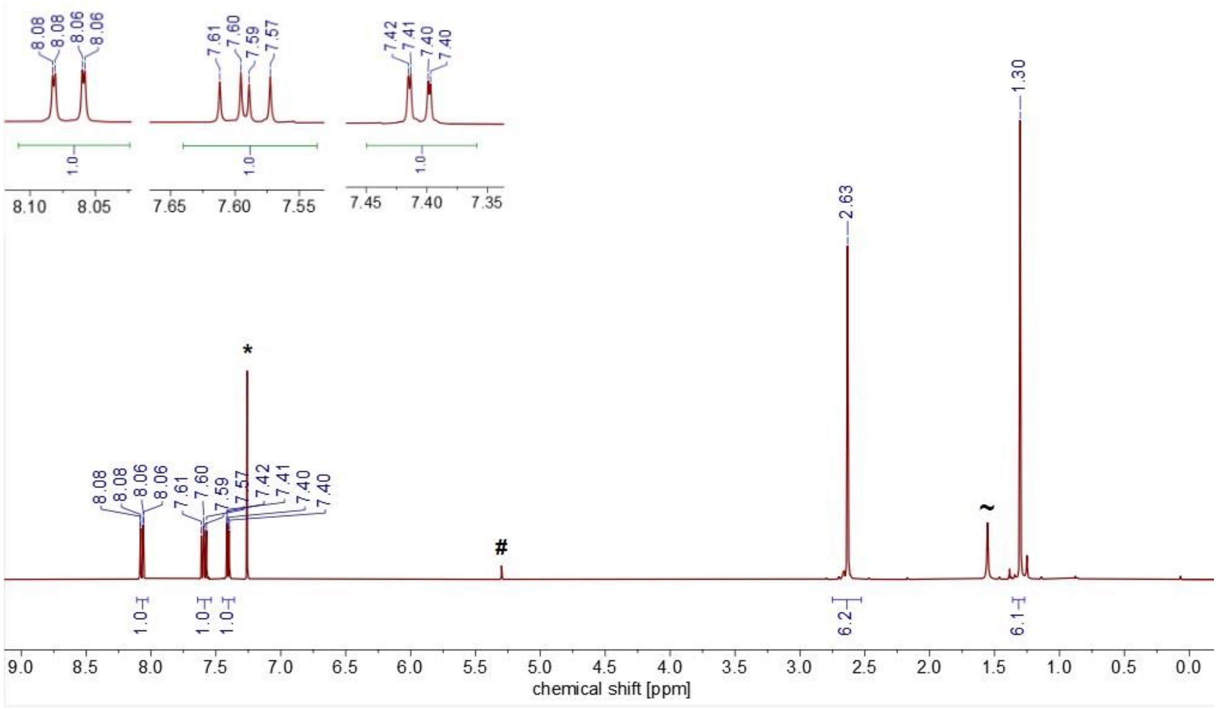

Figure S4. ^{1}H NMR of BODIPY **2a** in deuterated chloroform. The symbols *, #, and ~ indicate chloroform, dichloromethane, and water residual peaks, respectively.

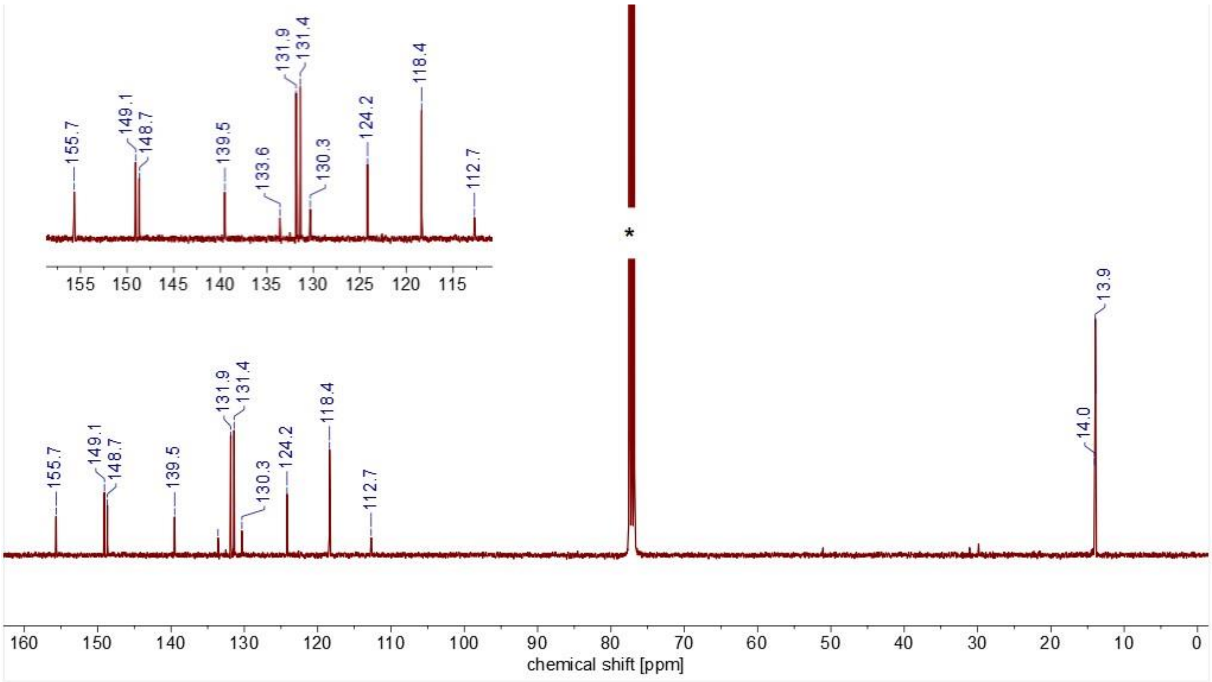

Figure S5. ^{13}C NMR of BODIPY **2a**. The symbols * indicate chloroform residual peak.

Table S2. NMR data for BODIPY **2a,** including key correlations determined from ^{1}H-^{1}H COSY, ^{1}H-^{13}C HSQC, and ^{1}H-^{13}C HMBC spectra.

**Proton δ (ppm)**	**Proton J (*Hz*)**	**^{1}H – ^{1}H COSY δ (ppm)**	**Carbon atom attached δ (ppm)**	**1H-13C HMBC δ (ppm)**
1.30	s	2.63	13.9	112.7; 130.3; 139.5; 155.7
2.63	s	1.30	14.0	112.7; 139.5; 155.7
7.41	dd (6.6; 0.8)	7.59	131.9	118.4; 133.6; 148.7
7.59	dd (9.1; 6.6)	7.41; 8.07	131.4	124.2; 131.9; 149.1
8.07	dd (9.1; 0.8)	7.59	118.4	124.2; 131.4; 148.7

Carbon signals [ppm]: 13.9; 14.0; 112.7; 118.4; 124.2; 130.3; 131.4; 131.9; 133.6; 139.5; 148.7; 149.1; 155.7.

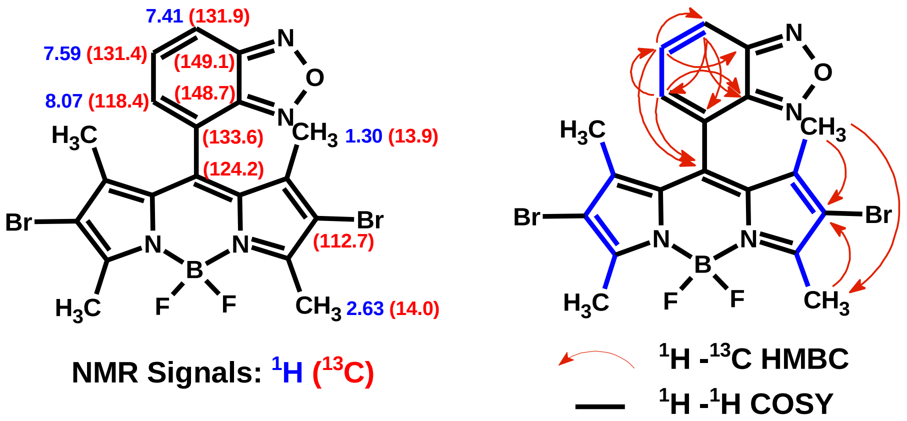

Figure S6. ^{1}H and (^{13}C) chemical shift values [ppm] and key correlations observed in NMR spectra of **2b**.

Compound 2b

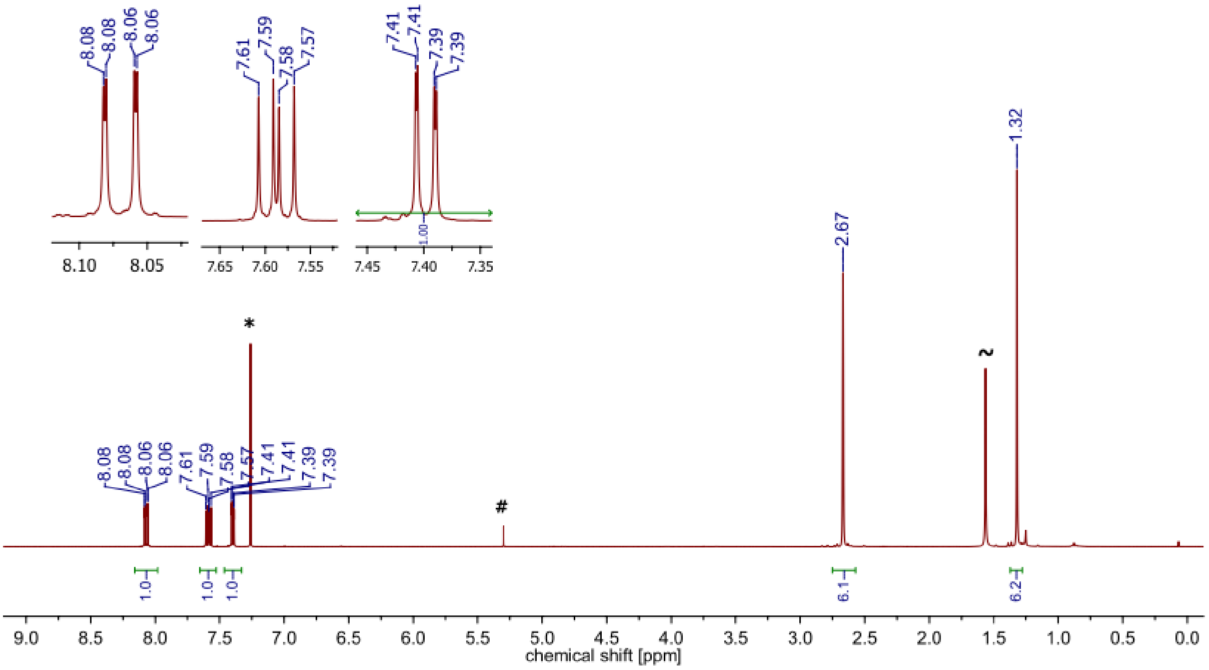

Figure S7. ^{1}H NMR of BODIPY **2b** in deuterated chloroform. The symbols *, #, and ~ indicate chloroform, dichloromethane, and water residual peaks, respectively.

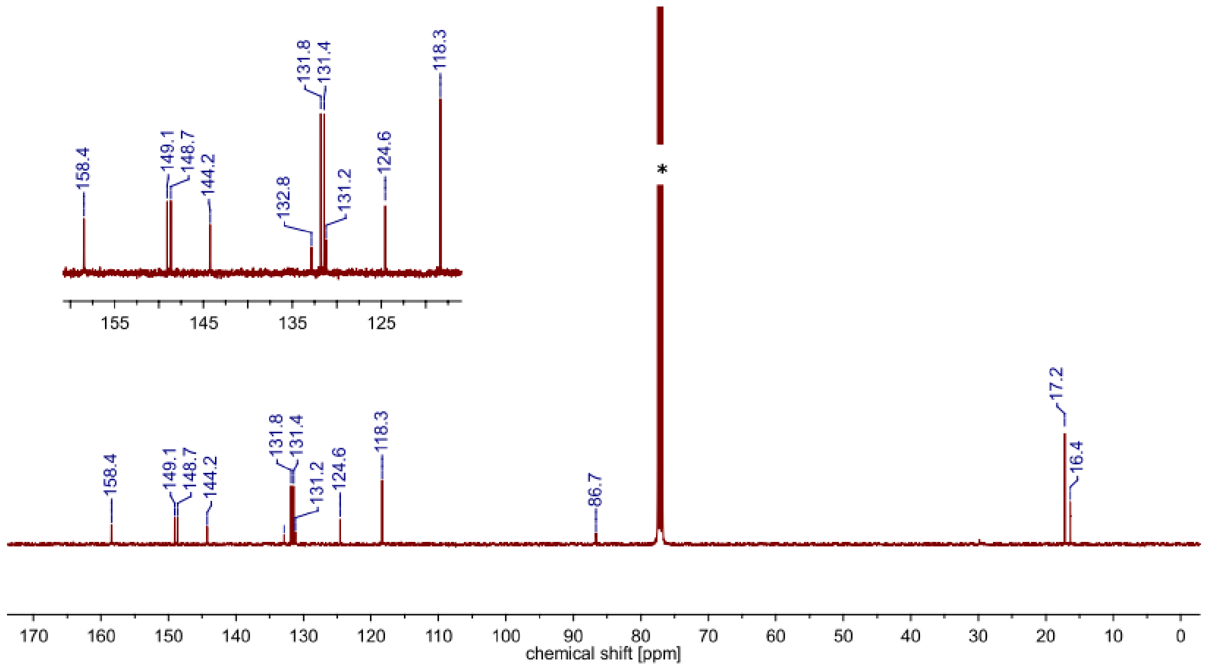

Figure S8. ^{13}C NMR of BODIPY **2b**. The symbol * indicates chloroform residual peak.

Table S3. NMR data for **2b,** including key correlations determined from ^{1}H-^{1}H COSY, ^{1}H-^{13}C HSQC, and ^{1}H-^{13}C HMBC spectra.

Proton δ (ppm)	Proton J (*Hz*)	^{1}H – ^{1}H COSY δ (ppm)	Carbon atom attached δ (ppm)	1H-13C HMBC δ (ppm)
1.32	s	2.67	17.2	16.4; 86.7; 131.2; 144.2; 158.4
2.67	s	1.32	16.4	86.7; 132.8; 144.2; 158.4
7.40	dd (6.6; 0.8)	7.59	131.8	118.3; 132.8; 148.7
7.59	dd (9.1; 6.6)	7.40; 8.07	131.4	124.6; 131.8; 149.1
8.07	dd (9.1; 0.8)	7.59	118.3	124.6; 131.8; 148.7
Carbon signals: 16.4; 17.2; 86.7; 118.3; 124.6; 131.2; 131.4; 131.8; 132.8; 144.2; 148.7; 149.1; 158.4.				

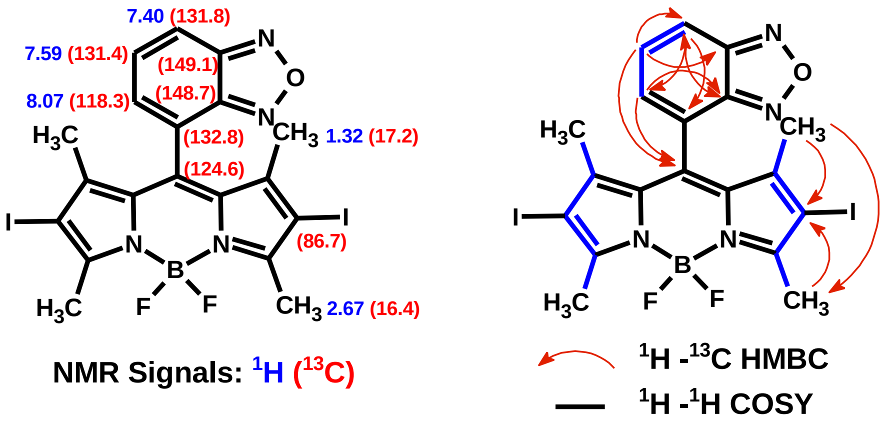

Figure S9. ^{1}H and (^{13}C) chemical shift values [ppm] and key correlations observed in NMR spectra of **2b**.

Mass Spectrometry data

Acquisition Parameter

Source Type	ESI	Ion Polarity	Positive	Set Nebulizer	0.3 Bar
Focus	Active	Set Capillary	4200 V	Set Dry Heater	200 °C
Scan Begin	100 m/z	Set End Plate Offset	-500 V	Set Dry Gas	4.0 l/min
Scan End	1100 m/z	Set Charging Voltage	2000 V	Set Divert Valve	Source
		Set Corona	0 nA	Set APCI Heater	0 °C

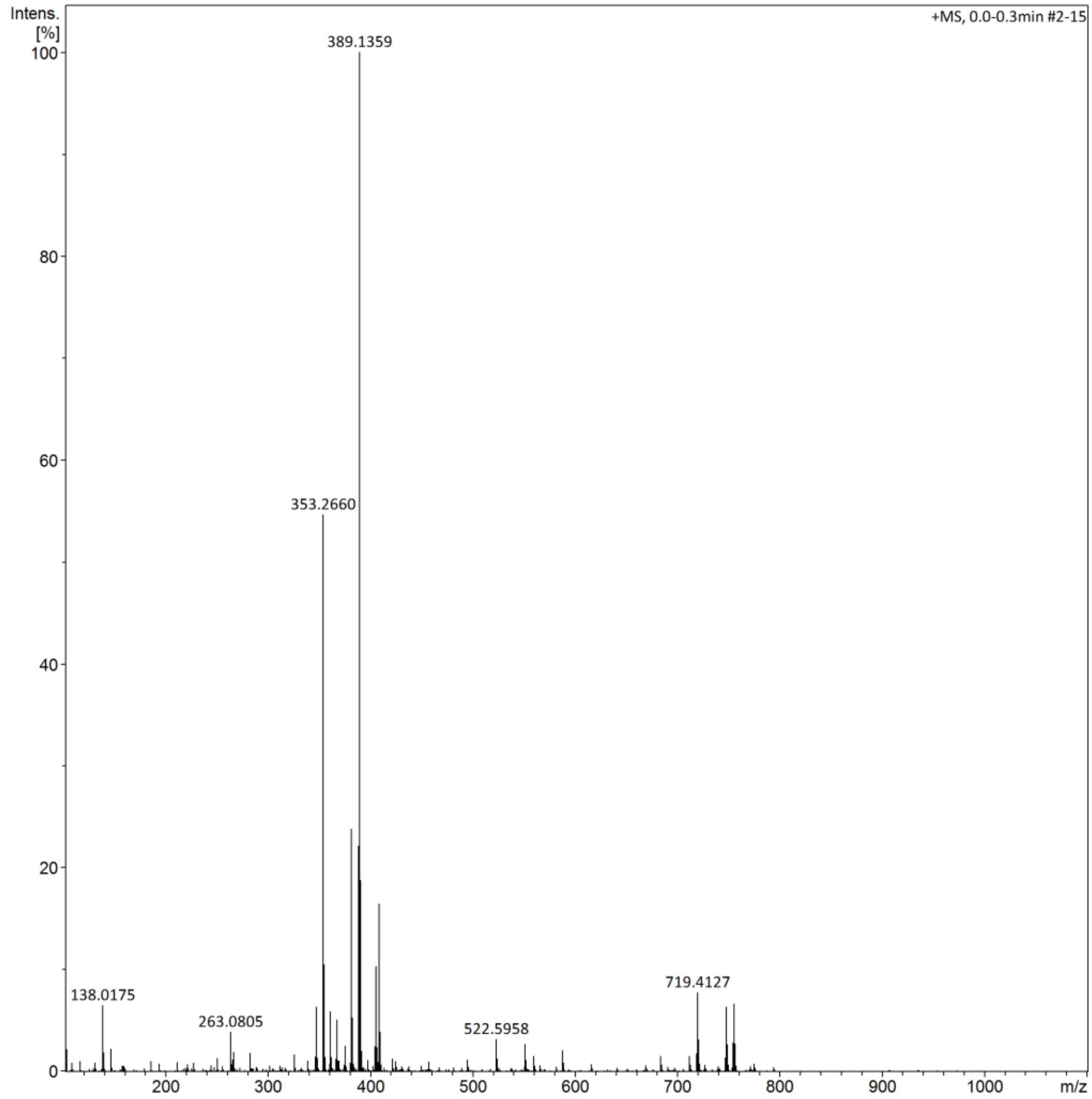

Acquisition Parameter

Source Type	ESI	Ion Polarity	Positive	Set Nebulizer	0.3 Bar
Focus	Active	Set Capillary	4200 V	Set Dry Heater	200 °C
Scan Begin	100 m/z	Set End Plate Offset	-500 V	Set Dry Gas	4.0 l/min
Scan End	1100 m/z	Set Charging Voltage	2000 V	Set Divert Valve	Source
		Set Corona	0 nA	Set APCI Heater	0 °C

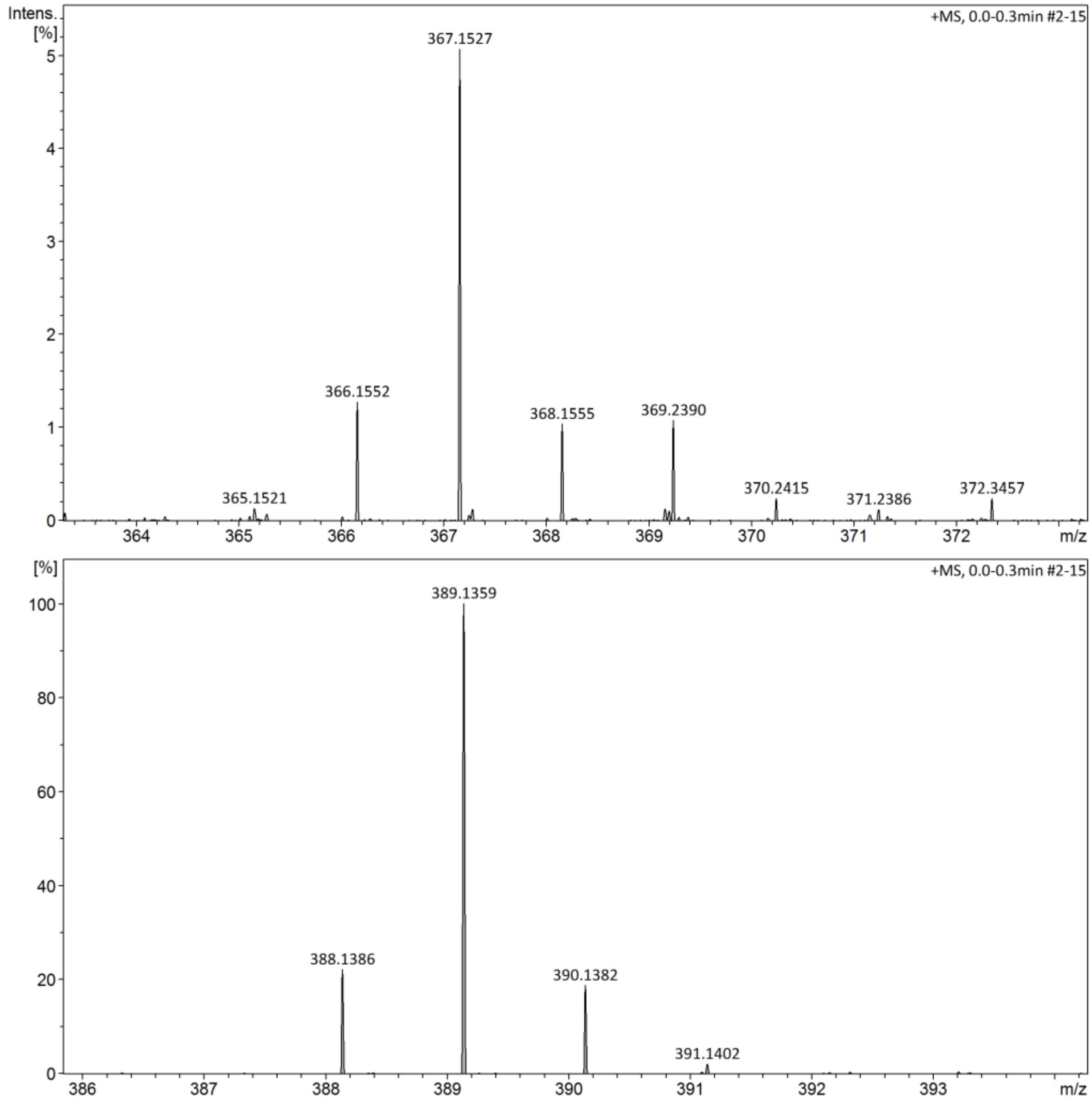

Figure S10. HRMS spectra of BODIPY **1**.

Acquisition Parameter

Source Type	ESI	Ion Polarity	Positive	Set Nebulizer	0.3 Bar
Focus	Active	Set Capillary	4200 V	Set Dry Heater	200 °C
Scan Begin	400 m/z	Set End Plate Offset	-500 V	Set Dry Gas	4.0 l/min
Scan End	1100 m/z	Set Charging Voltage	2000 V	Set Divert Valve	Source
		Set Corona	0 nA	Set APCI Heater	0 °C

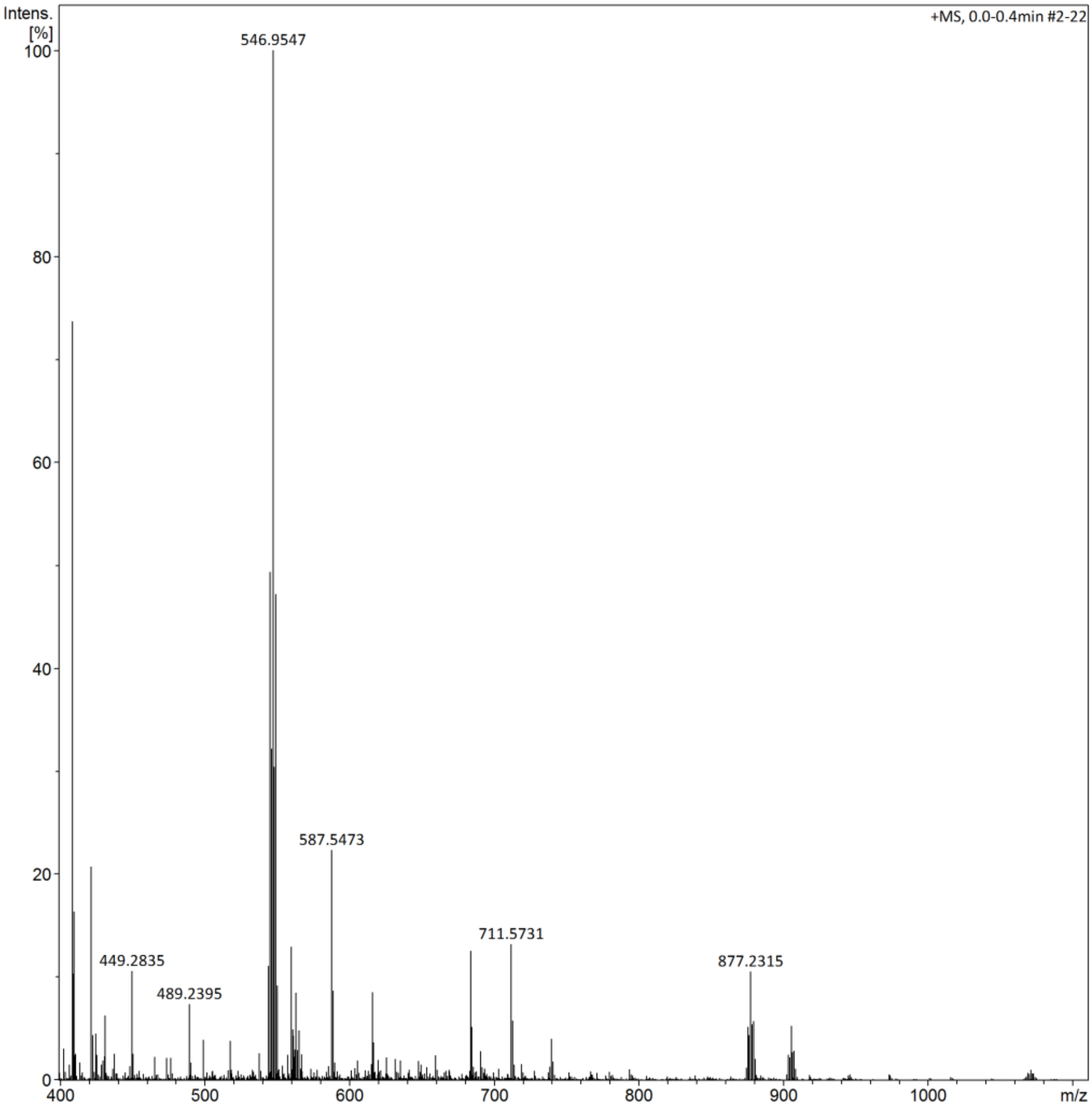

Acquisition Parameter

Source Type	ESI	Ion Polarity	Positive	Set Nebulizer	0.3 Bar
Focus	Active	Set Capillary	4200 V	Set Dry Heater	200 °C
Scan Begin	400 m/z	Set End Plate Offset	-500 V	Set Dry Gas	4.0 l/min
Scan End	1100 m/z	Set Charging Voltage	2000 V	Set Divert Valve	Source
		Set Corona	0 nA	Set APCI Heater	0 °C

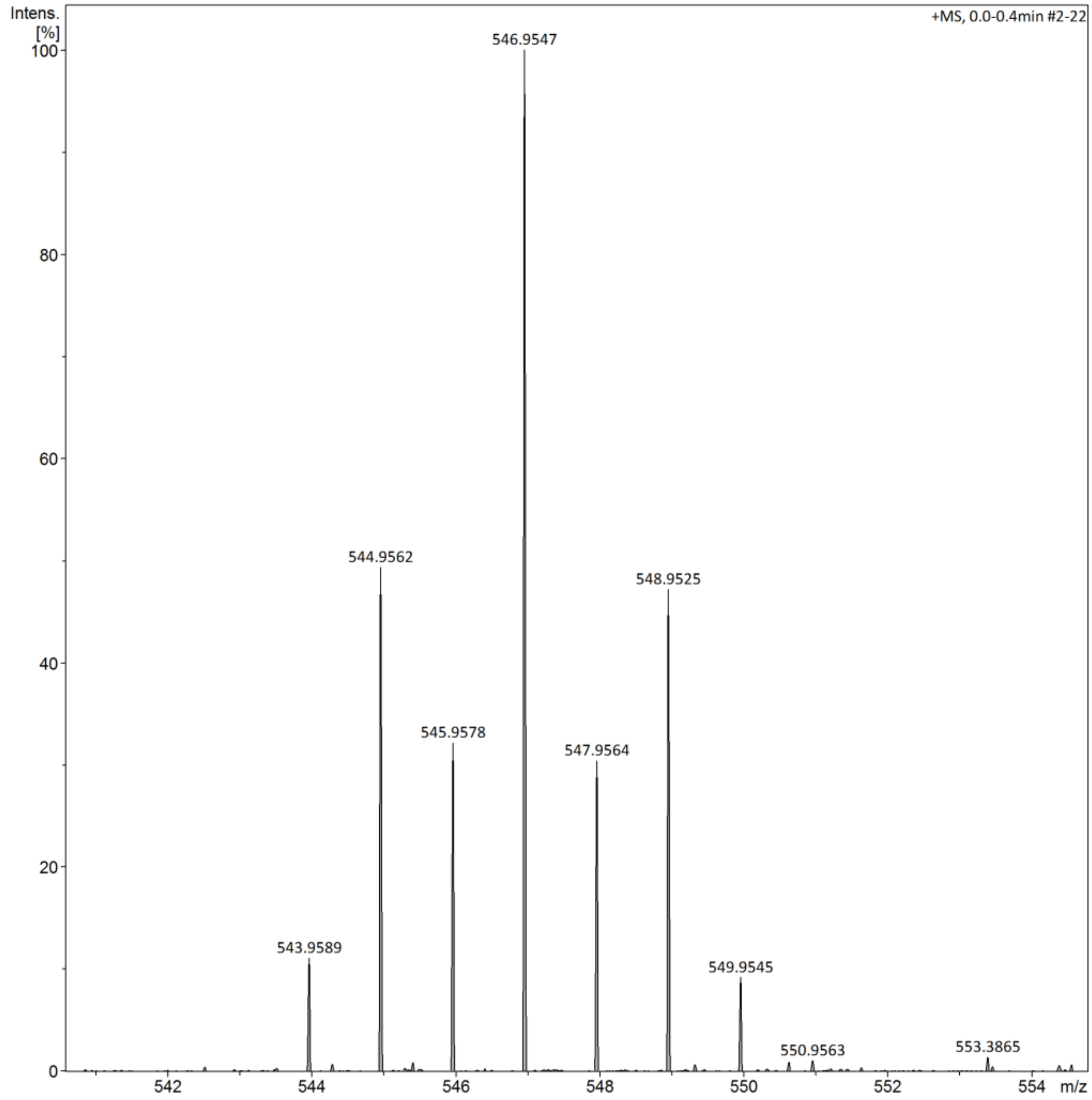

Figure S11. HRMS spectra of BODIPY **2a**.

Acquisition Parameter

Source Type	ESI	Ion Polarity	Positive	Set Nebulizer	0.3 Bar
Focus	Active	Set Capillary	4200 V	Set Dry Heater	200 °C
Scan Begin	430 m/z	Set End Plate Offset	-500 V	Set Dry Gas	4.0 l/min
Scan End	1100 m/z	Set Charging Voltage	2000 V	Set Divert Valve	Source
		Set Corona	0 nA	Set APCI Heater	0 °C

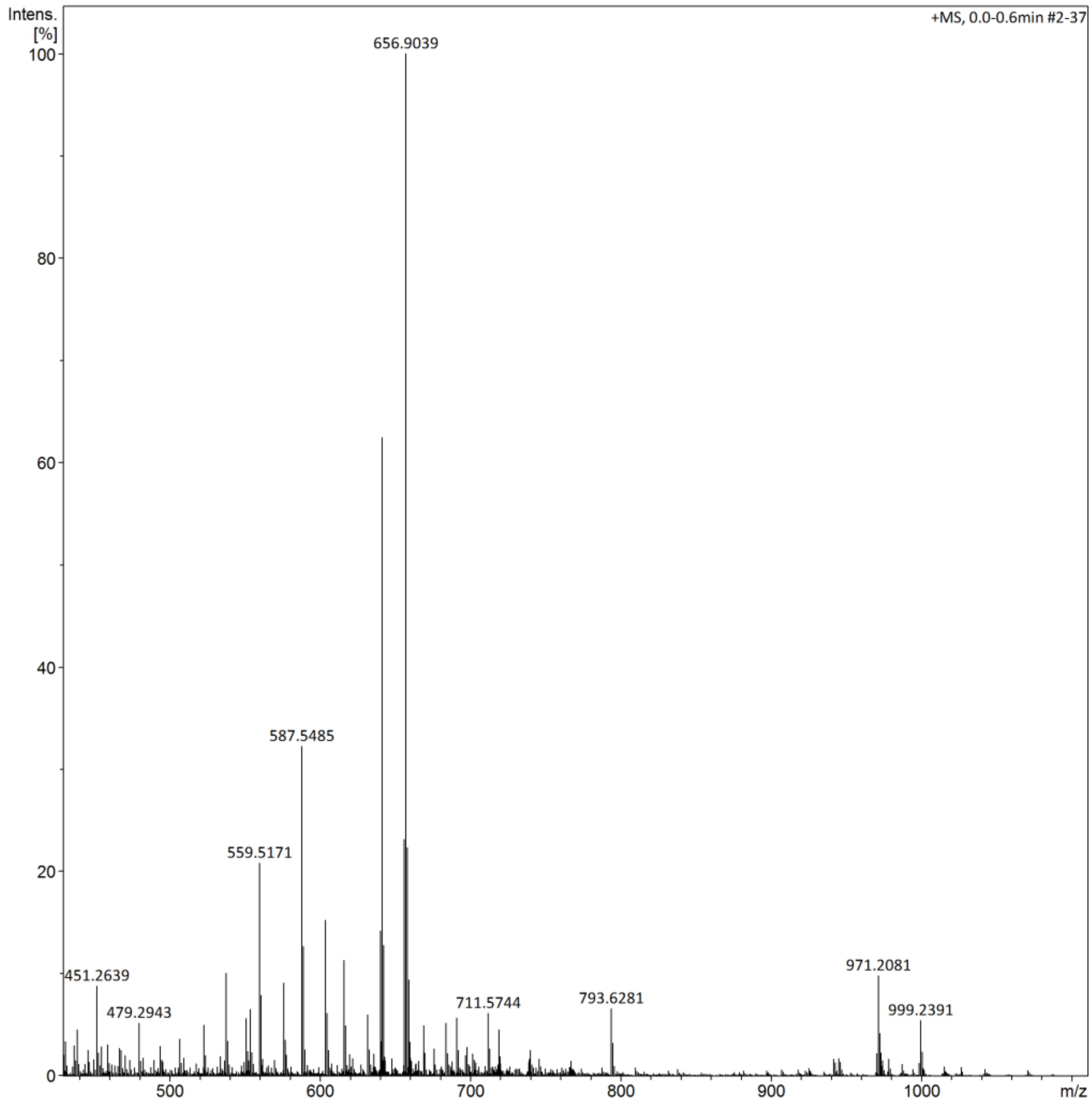

Acquisition Parameter

Source Type	ESI	Ion Polarity	Positive	Set Nebulizer	0.3 Bar
Focus	Active	Set Capillary	4200 V	Set Dry Heater	200 °C
Scan Begin	430 m/z	Set End Plate Offset	-500 V	Set Dry Gas	4.0 l/min
Scan End	1100 m/z	Set Charging Voltage	2000 V	Set Divert Valve	Source
		Set Corona	0 nA	Set APCI Heater	0 °C

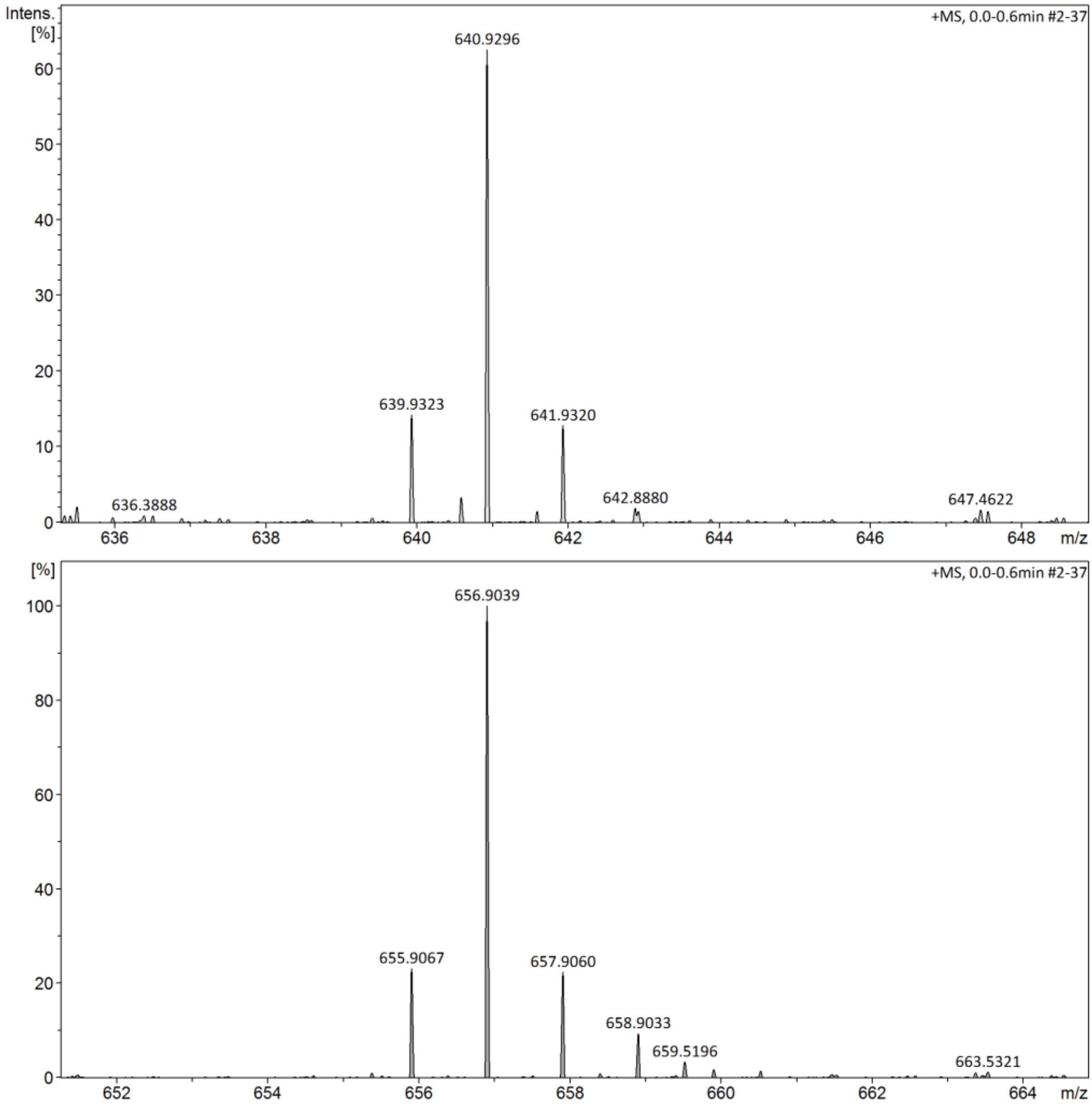

Figure S12. HRMS spectra of BODIPY **2b**.

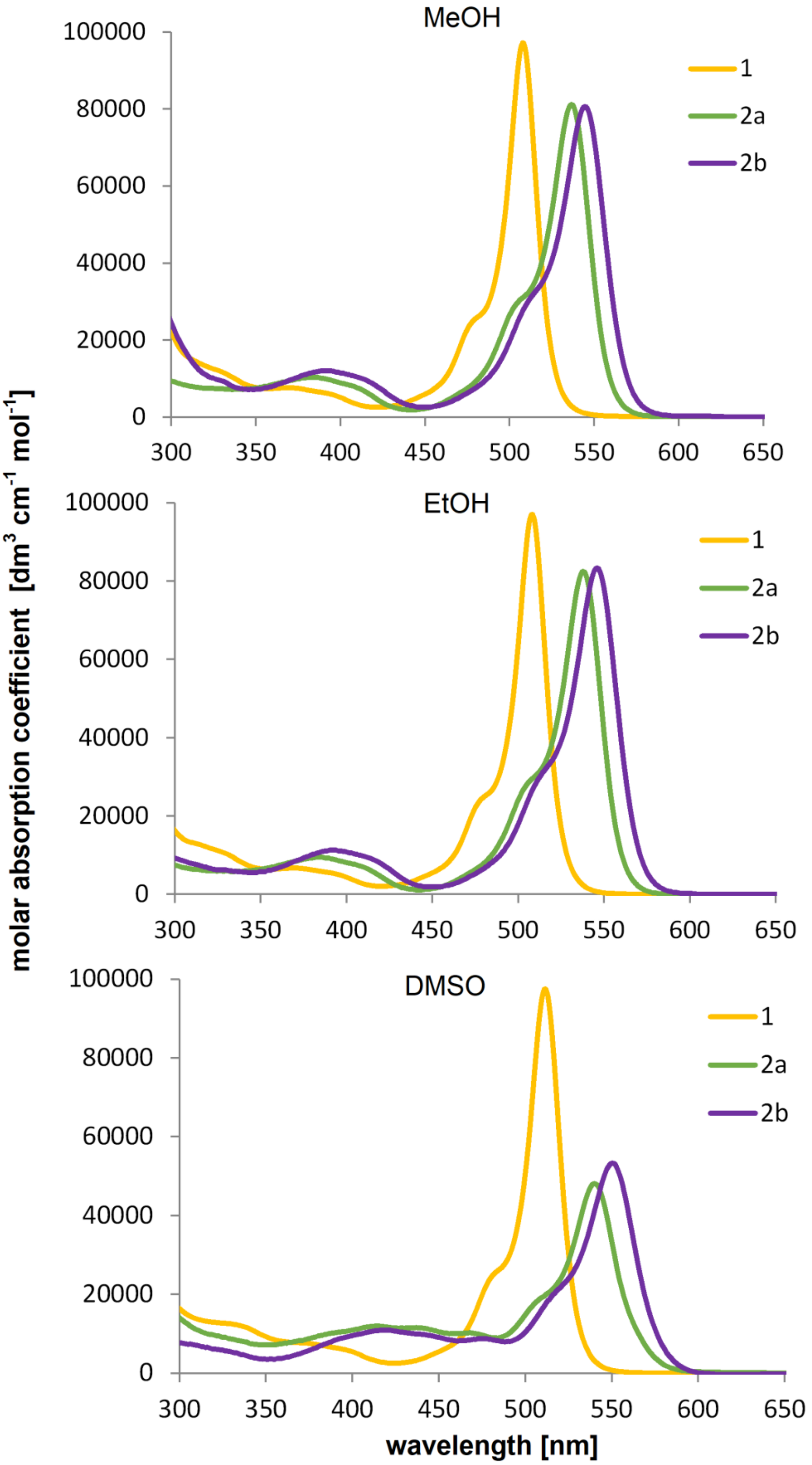

Figure S13. Absorption spectra of compounds **1, 2a,** and **2b**.

Table S4. UV-Vis absorption maxima (λ_{Abs}) and logarithms of molar absorption coefficients (log ε ± SD) of compounds **1, 2a,** and **2b** in various solvents.

		BODIPY			
compound	solvent	**DCM**	**MeOH**	**EtOH**	**DMSO**
**1**	λ_{Abs} (log ε)	512 (4.98±0.04)	508 (4.99±0.04)	509 (4.99±0.04)	511 (4.99±0.03)
**2a**	λ_{Abs} (log ε)	542 (4.91±0.03)	537 (4.91±0.03)	538 (4.92±0.03)	540 (4.67±0.11)
**2b**	λ_{Abs} (log ε)	549 (4.94±0.05)	545 (4.90±0.05)	546 (4.92±0.05)	550 (4.71±0.10)

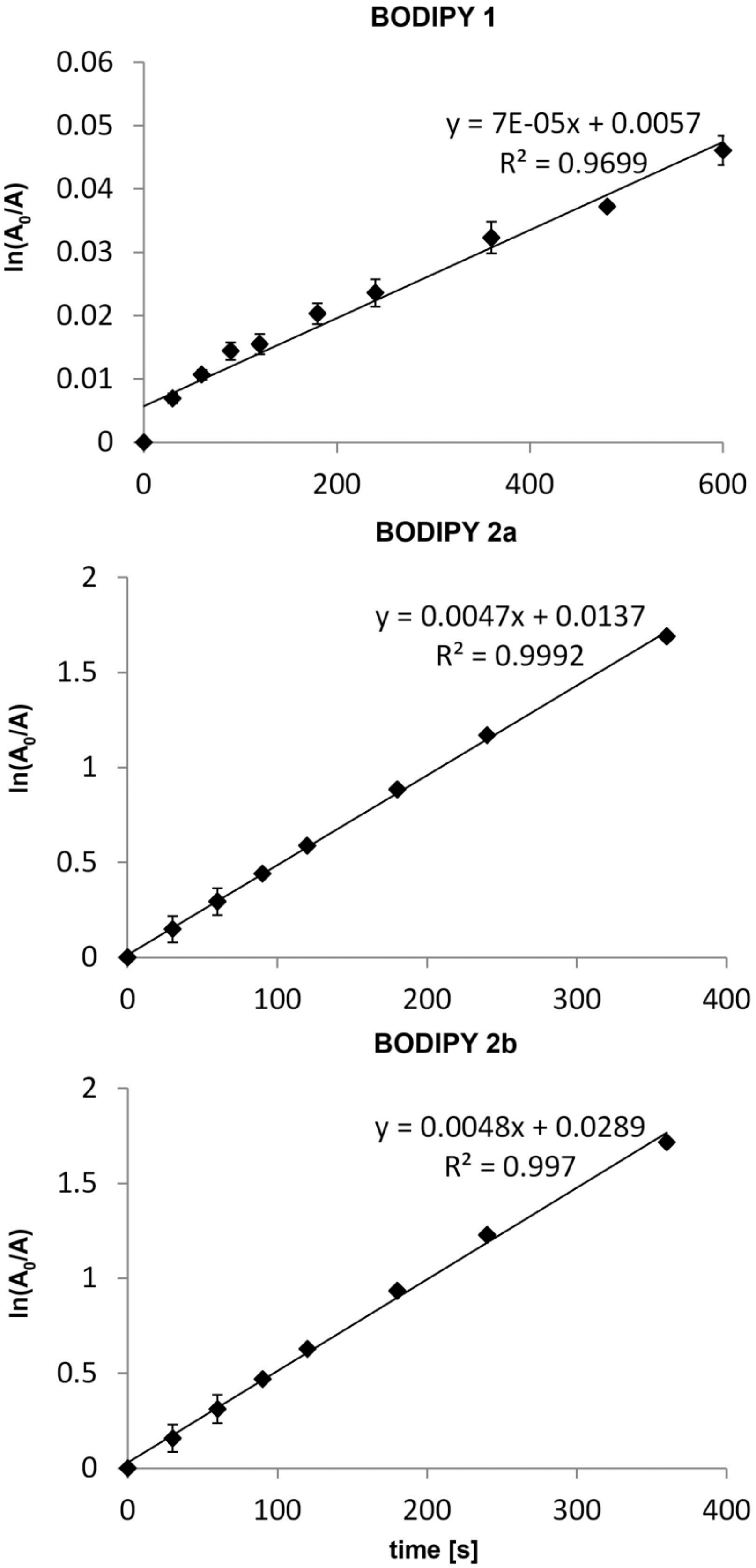

Figure S14. The first-order plots for the oxidation of the DPBF for BODIPYs **1**, **2a,** and **2b** in methanol.

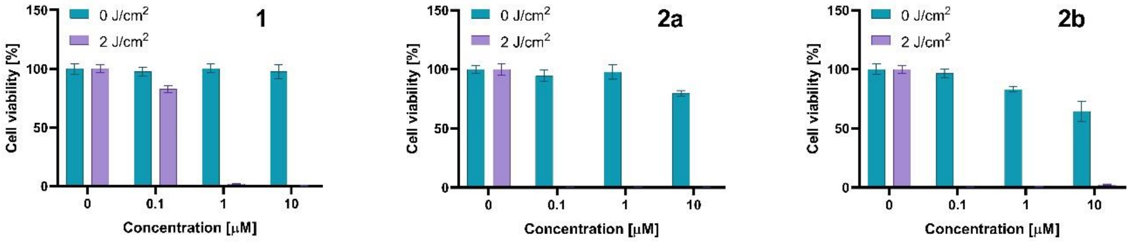

Figure S15. The preliminary results for compounds **1**, **2a**, and **2b** towards MDA-MB-231 cells performed under normoxic conditions (screening experiment).

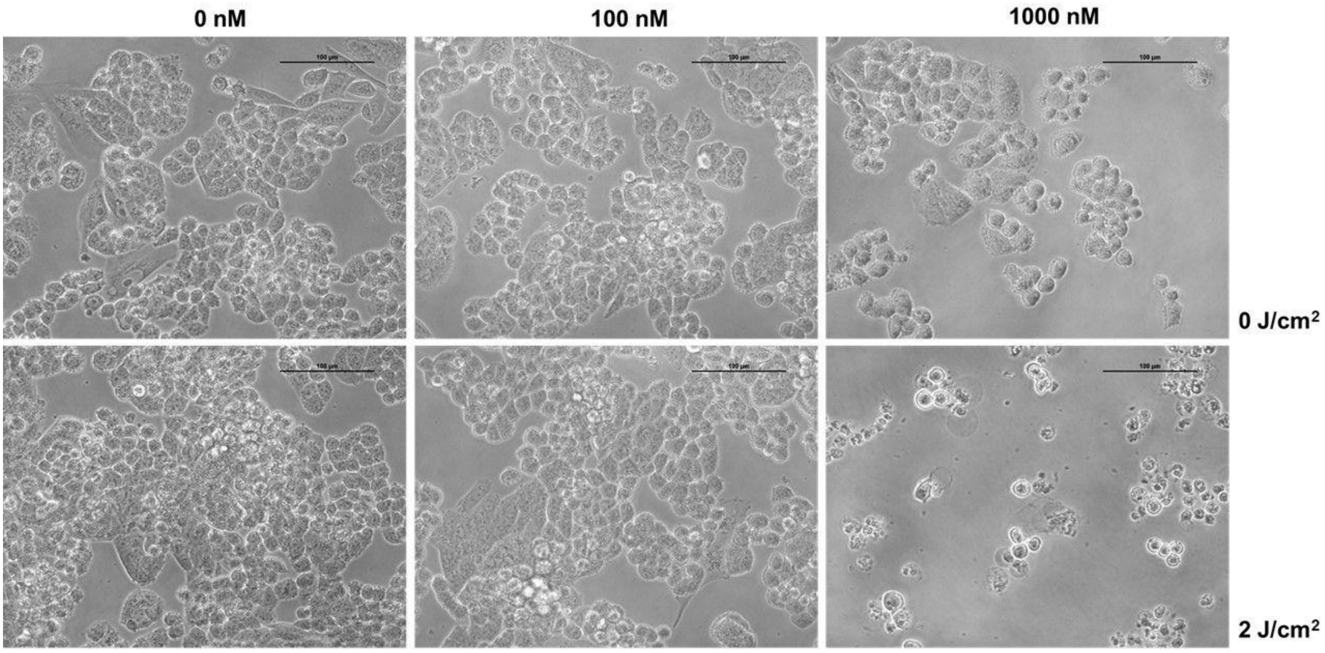

Figure S16. The A2780 morphology after treatment with BODIPY **1**. The images were taken with a DS-SMc digital camera attached to a Nikon Eclipse TS100 microscope. The scale bar corresponds to 100 μm.

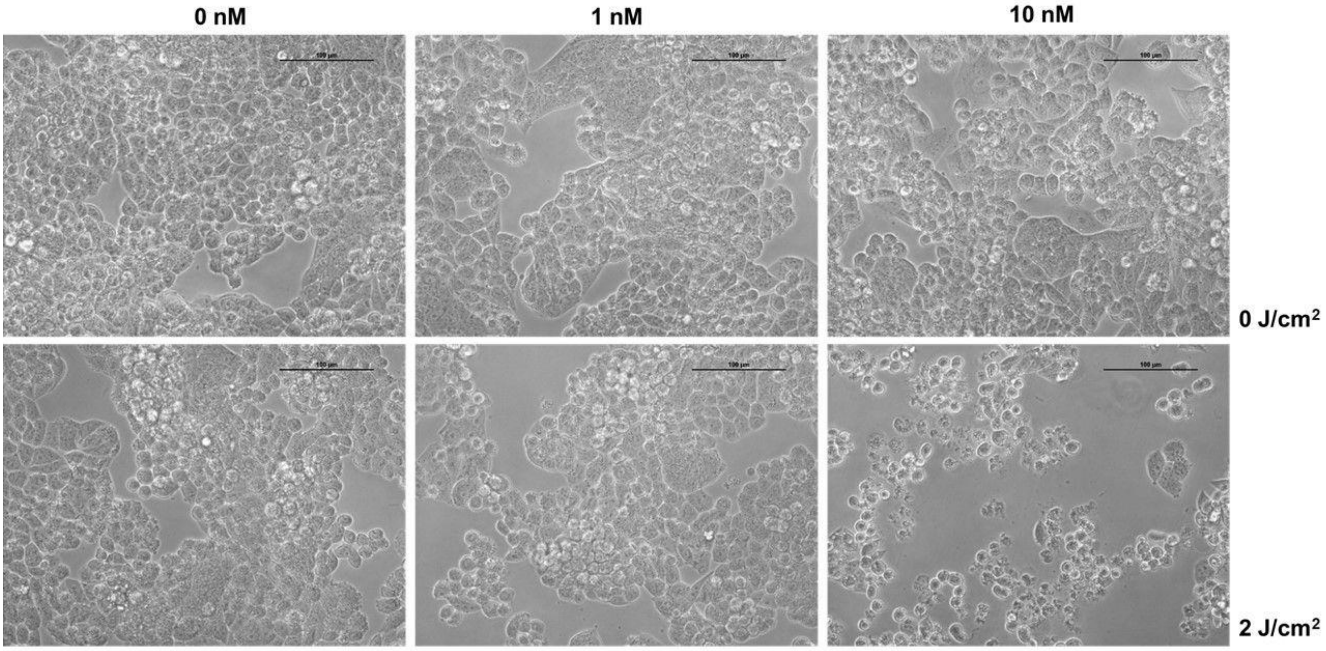

Figure S17. The A2780 cells morphology after treatment with BODIPY **2a**. The images were taken with a DS-SMc digital camera attached to a Nikon Eclipse TS100 microscope. The scale bar corresponds to 100 μm.

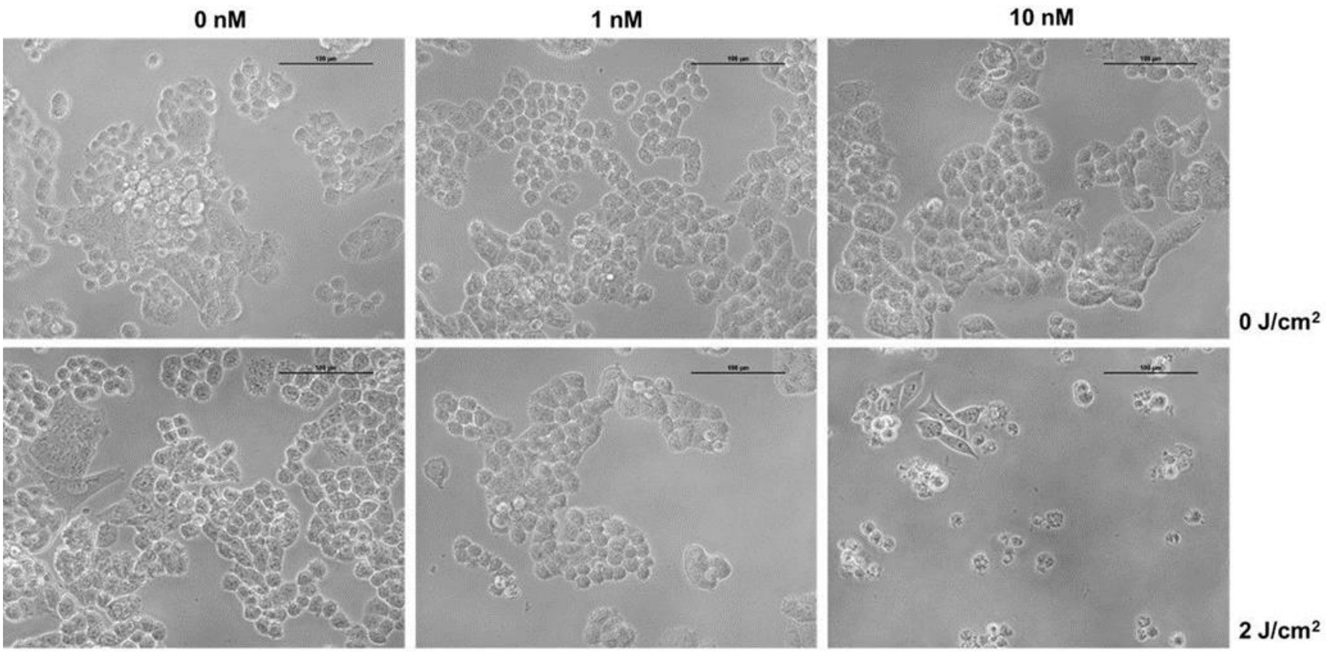

Figure S18. The A2780 morphology after treatment with BODIPY **2b**. The images were taken with a DS-SMc digital camera attached to a Nikon Eclipse TS100 microscope. The scale bar corresponds to 100 μm.

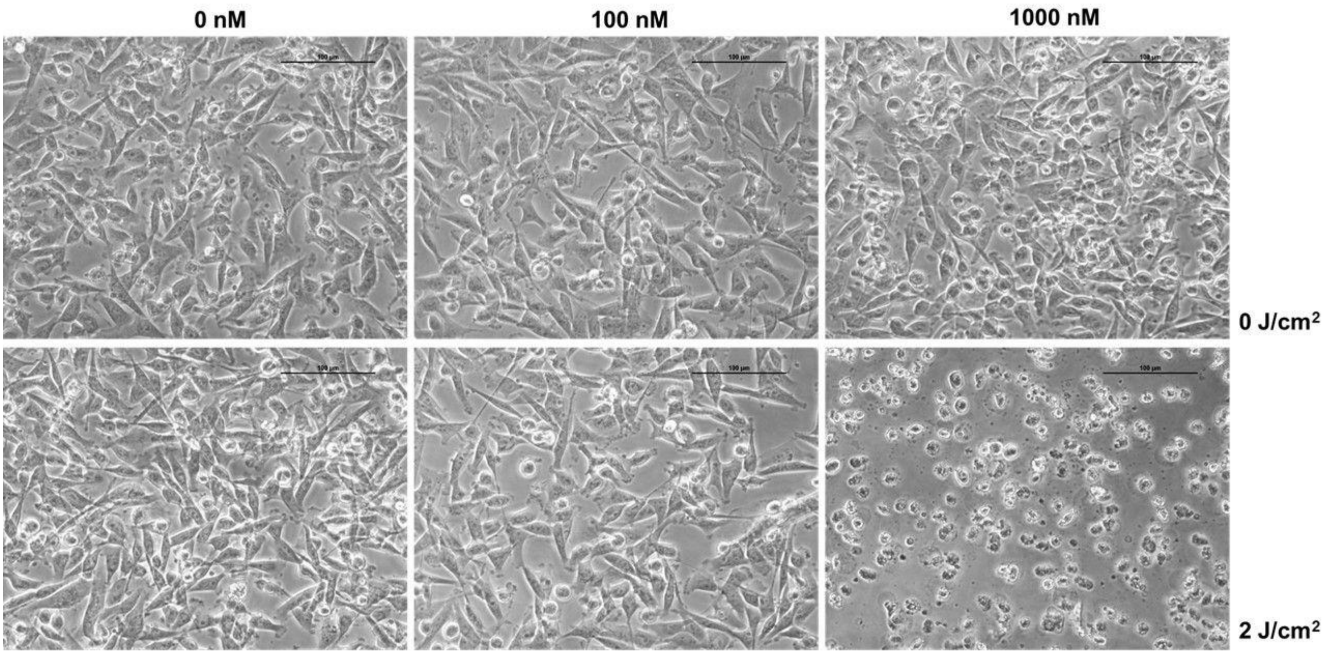

Figure S19. The MDA-MB-231 cells morphology after treatment with BODIPY **1**. The images were taken with a DS-SMc digital camera attached to a Nikon Eclipse TS100 microscope. The scale bar corresponds to 100 μm.

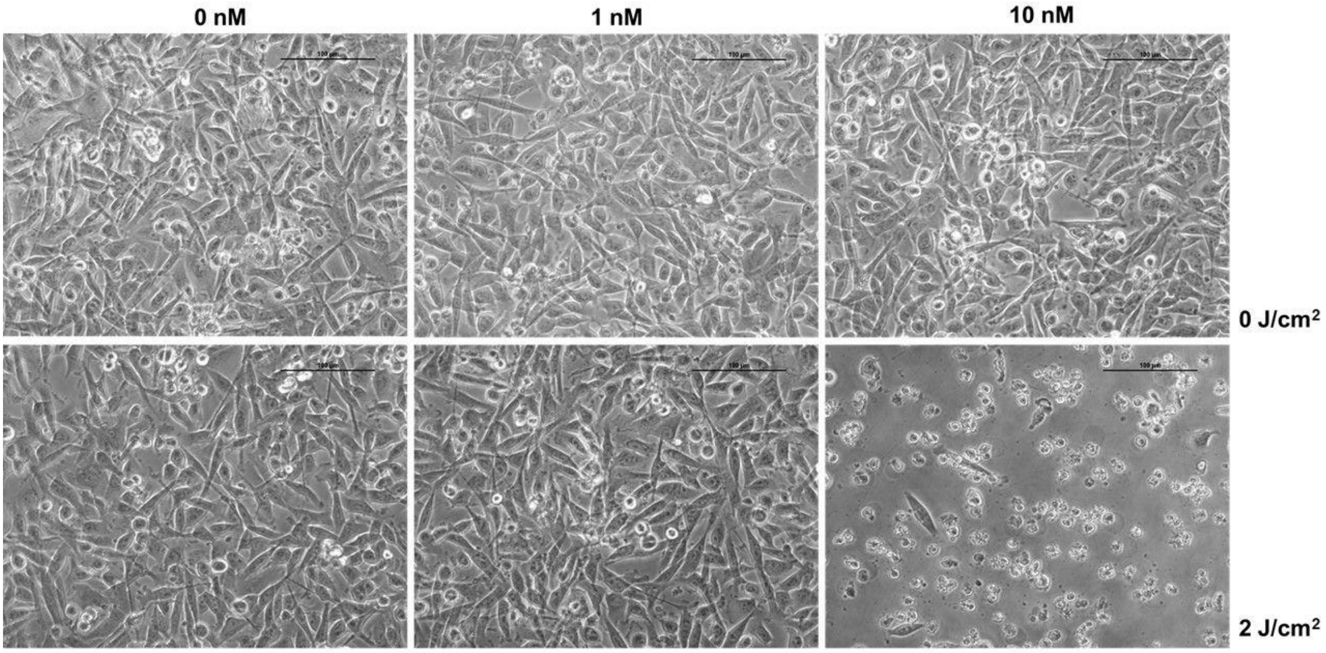

Figure S20. The MDA-MB-231 cells morphology after treatment with BODIPY **2a**. The images were taken with a DS-SMc digital camera attached to a Nikon Eclipse TS100 microscope. The scale bar corresponds to 100 μm.

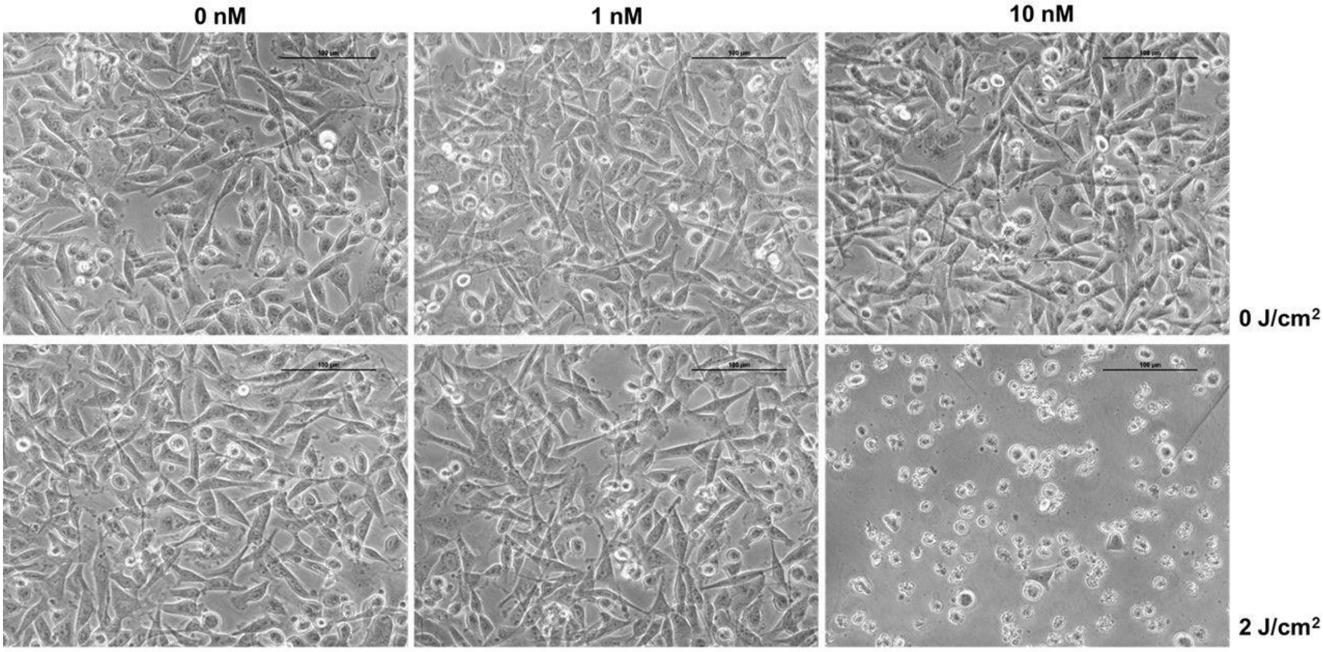

Figure S21. The MDA-MB-231 cells morphology after treatment with BODIPY **2b**. The images were taken with a DS-SMc digital camera attached to a Nikon Eclipse TS100 microscope. The scale bar corresponds to 100 μm.

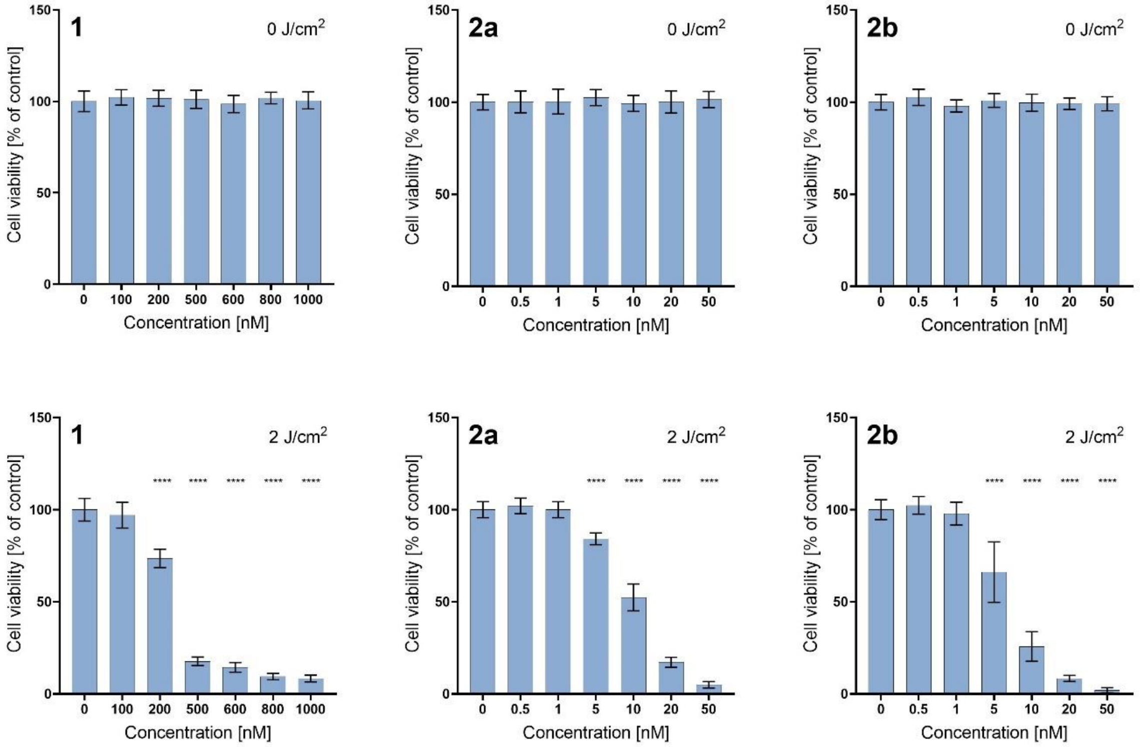

Figure S22. The viability of MDA-MB-231 cells after treatment with BODIPYs **1**, **2a**, and **2b** under normoxic conditions. Cell viability was measured using the MTT assay 24 h after irradiation (phototoxicity) or without irradiation (dark toxicity) under normoxic conditions. Data are presented as mean values ± SD, calculated from three independent experiments (except for compound 1 under hypoxic conditions, which was repeated once due to its lack of cytotoxic effect. Asterisks indicate statistical significance, **** $p < 0.0001$ vs. the control group. Statistical significance was measured using one-way ANOVA with Dunnett's multiple comparison tests.

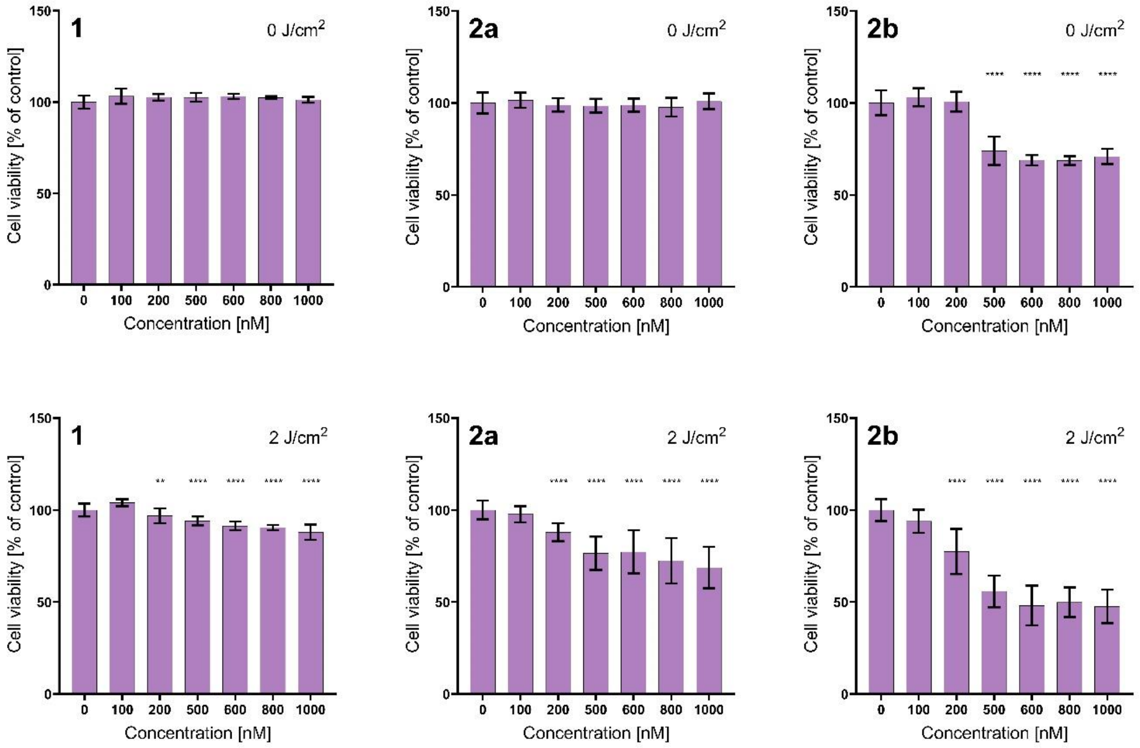

Figure S23. The viability of MDA-MB-231 cells after treatment with BODIPYs **1**, **2a**, and **2b** under hypoxic conditions. Cell viability was measured using the MTT assay 24 h after irradiation (phototoxicity) or without irradiation (dark toxicity). Data are presented as mean values ± SD, calculated from three independent experiments (except for compound **1** under hypoxic conditions, which was repeated once due to its lack of cytotoxic effect. Asterisks indicate statistical significance, ** $p < 0.01$; **** $p < 0.0001$ vs. the control group. Statistical significance was determined using one-way ANOVA followed by Dunnett's multiple comparisons test.

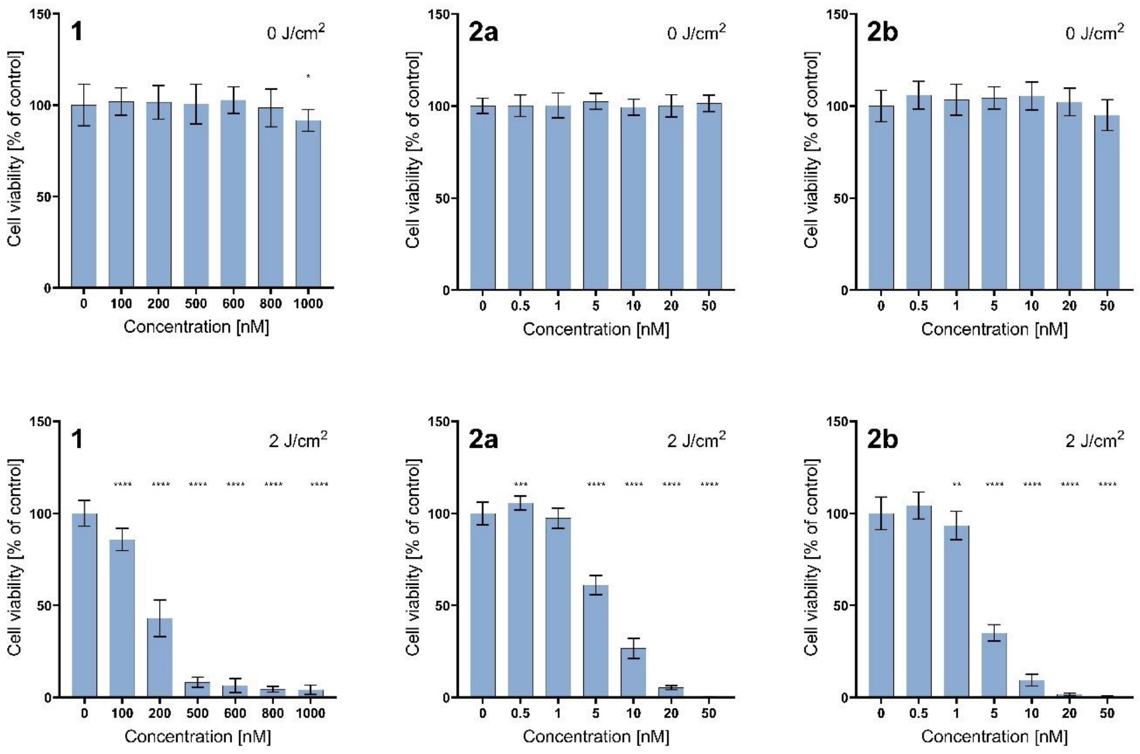

Figure S24. The viability of A2780 cells after treatment with BODIPYs **1**, **2a**, and **2b** under normoxic conditions. Cell viability was measured using the MTT assay 24 h after irradiation (phototoxicity) or without irradiation (dark toxicity). Data are presented as mean values ± SD, calculated from three independent experiments (except for compound **1** under hypoxic conditions, which was repeated once due to its lack of cytotoxic effect). Asterisks indicate statistical significance, * p<0.05; ** p< 0.01; *** p< 0.001; **** p < 0.0001 vs. the control group. Statistical significance was determined using one-way ANOVA followed by Dunnett's multiple comparisons test.

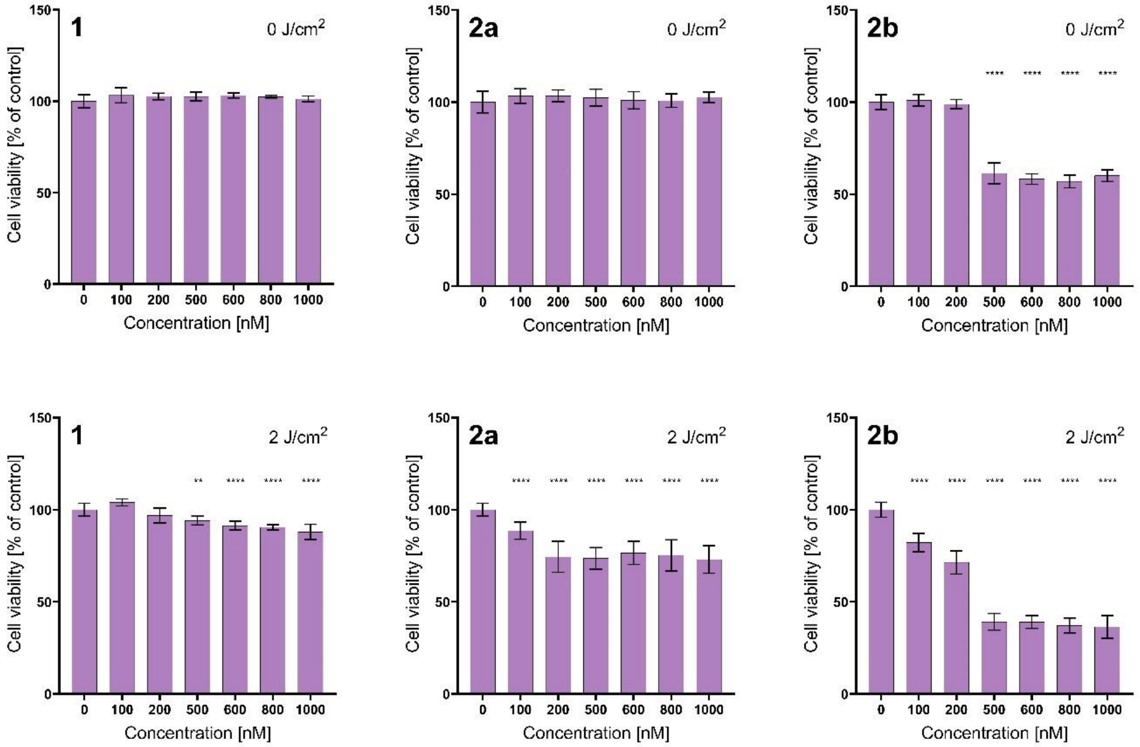

Figure S25. The viability of A2780 cells after treatment with BODIPYs **1**, **2a**, and **2b** under hypoxic conditions. Cell viability was measured using the MTT assay 24 h after irradiation (phototoxicity) or without irradiation (dark toxicity). Data are presented as mean values ± SD, calculated from three independent experiments (except for compound 1 under hypoxic conditions, which was repeated once due to its lack of cytotoxic effect). Asterisks indicate statistical significance, ** p< 0.01; **** p < 0.0001 vs. the control group. Statistical significance was determined using one-way ANOVA followed by Dunnett's multiple comparisons test.

Table S5. The cell viability of A2780 and MDA-MB-231 treated with **2a** and **2b** under hypoxic conditions without exposure to light.

		**A2780**			**MDA-MB-231**		
	**Concentration**	**Mean**	**SD**	**N**	**Mean**	**SD**	**N**
**2a**	0	100	5.86	36	100	5.73	36
	100	103.28	3.90	18	101.47	4.10	18
	200	103.33	3.19	18	98.93	3.63	18
	500	102.39	4.57	18	98.46	3.76	18
	600	100.93	4.71	18	98.76	3.61	18
	800	100.74	3.67	18	97.66	5.05	18
	1000	102.55	2.91	18	100.90	4.26	18
**2b**	0	100	4.074	36	100	6.77	36
	100	100.95	3.19	18	103.03	4.90	18
	200	98.82	3.38	18	100.65	5.35	18
	500	61.32	5.61	18	73.90	7.67	18
	600	58.17	2.80	18	68.78	2.84	18
	800	56.90	3.48	18	68.65	2.47	18
	1000	60.09	3.22	18	70.90	4.18	18

N- number of replicates (three independent experiments performed in six technical replicates).

Table S6. The cell viability of A2780 and MDA-MB-231 treated with **2a** and **2b** under hypoxic conditions after irradiation at a light dose of 2 J/cm^2.

		**A2780**			**MDA-MB-231**		
	**Concentration**	**Mean**	**SD**	**N**	**Mean**	**SD**	**N**
**2a**	0	100	3.51	36	100	5.074	36
	100	88.71	4.70	18	97.74	4.36	18
	200	74.46	8.46	18	87.90	4.87	18
	500	73.63	5.81	18	76.50	9.10	18
	600	76.58	6.23	18	77.31	11.68	18
	800	75.20	8.43	18	72.46	12.30	18
	1000	73.05	7.56	18	68.70	11.27	18
**2b**	0	100	4.048	36	100	5.97	36
	100	82.16	4.50	18	93.93	6.34	18
	200	71.41	6.29	18	77.49	12.29	18
	500	39.14	4.49	18	55.76	8.60	18
	600	39.06	3.44	18	48.13	10.81	18
	800	37.22	4.05	18	49.88	8.10	18
	1000	36.50	6.16	18	47.65	9.05	18

N- number of replicates (three independent experiments performed in six technical replicates).
